# De Novo Gene Birth, Horizontal Gene Transfer, and Gene Duplication as Sources of New Gene Families Associated with the Origin of Symbiosis in *Amanita*

**DOI:** 10.1093/gbe/evaa193

**Published:** 2020-09-14

**Authors:** Yen-Wen Wang, Jaqueline Hess, Jason C Slot, Anne Pringle

**Affiliations:** e1 Departments of Botany and Bacteriology, University of Wisconsin-Madison; e2 Department of Soil Ecology, Helmholtz Centre for Environmental Research - UFZ, Leipzig, Germany; e3 Department of Plant Pathology, The Ohio State University

**Keywords:** convergent evolution, gene family evolution, mycorrhizal fungi, phylogenomics, mutualism

## Abstract

By introducing novel capacities and functions, new genes and gene families may play a crucial role in ecological transitions. Mechanisms generating new gene families include de novo gene birth, horizontal gene transfer, and neofunctionalization following a duplication event. The ectomycorrhizal (ECM) symbiosis is a ubiquitous mutualism and the association has evolved repeatedly and independently many times among the fungi, but the evolutionary dynamics enabling its emergence remain elusive. We developed a phylogenetic workflow to first understand if gene families unique to ECM *Amanita* fungi and absent from closely related asymbiotic species are functionally relevant to the symbiosis, and then to systematically infer their origins. We identified 109 gene families unique to ECM *Amanita* species. Genes belonging to unique gene families are under strong purifying selection and are upregulated during symbiosis, compared with genes of conserved or orphan gene families. The origins of seven of the unique gene families are strongly supported as either de novo gene birth (two gene families), horizontal gene transfer (four), or gene duplication (one). An additional 34 families appear new because of their selective retention within symbiotic species. Among the 109 unique gene families, the most upregulated gene in symbiotic cultures encodes a 1-aminocyclopropane-1-carboxylate deaminase, an enzyme capable of downregulating the synthesis of the plant hormone ethylene, a common negative regulator of plant-microbial mutualisms.

**Significance**

Mutualisms between fungi and plants appear complex but have evolved repeatedly and independently many times. This convergent evolution is typically explained by gene loss from fungi, but at the origin of a symbiosis new genes also appear in fungi: where do these new genes come from and what do they do? By systematically querying the origin of genes unique to symbiotic *Amanita* fungi and not found in close relatives we discover de novo gene birth, horizontal gene transfer, and gene duplication as the sources of a set of highly selected new genes upregulated during symbiosis.

## Introduction

**Fig. 1.**
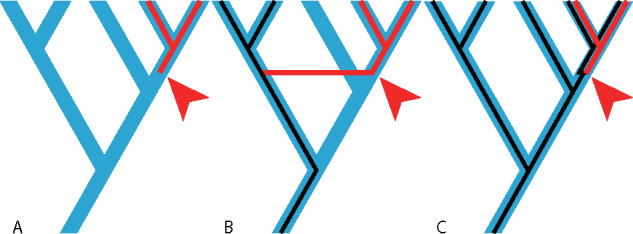
Three mechanisms of gene family emergence. (*A*) de novo gene birth; (*B*) HGT; (*C*) gene duplication. Blue, species tree; Red, new gene families (arrow indicates origin); Black, homologous genes.

Evolutionary novelties are novel properties or features of organisms facilitating adaptation (Mayr 1963; Pigliucci 2008). The concept of an evolutionary novelty can connect dramatic changes in morphologies or phenotypes with ecological transitions in niche. New gene families, without apparent homologies in ancestors, may be considered as genetic evolutionary novelties because they are heritable features potentially shaping adaptations and niche transitions (Villanueva-Cañas et al. 2017). New gene families are thought to have three principal sources (Long et al. 2013; Andersson et al. 2015): de novo gene birth, horizontal gene transfer (HGT)and gene duplication (fig. 1). However, genes as evolutionary novelties remain understudied and the functions of many young gene families are unknown.

De novo gene birth involves the formation of protein-coding exons from ancestral noncoding loci. Newly evolved exons are typically shorter and bear weaker signatures of purifying selection compared with existing genes (Carvunis et al. 2012; Ruiz-Orera et al*.* 2018; Vakirlis et al. 2018). Often, de novo genes are identified by the absence of homologous genes in protein databases; hypothetical de novo genes are confirmed by aligning their DNA sequences against putatively homologous, noncoding sequences found in closely related species (Cai et al. 2008; Knowles and McLysaght 2009). A robust example of a de novo gene birth is the *BSC4* gene of *Saccharomyces cerevisiae*. Gene expression data suggest *BSC4* is not a pseudogene, and the sequence homologies between BSC4 and syntenic but noncoding regions in closely related species confirm the de novo nature of this gene (Cai *et al.* 2008).

As mechanisms mediating the emergence of evolutionary novelties, HGT and gene duplication are better understood compared with de novo gene birth. HGT genes are often identified when the topologies between a species phylogeny and the phylogeny of a putative HGT gene family are inconsistent (Keeling and Palmer 2008; Husnik and McCutcheon 2018). Because HGT typically involves the movement of genes into distantly related lineages, HGT genes may have distinct properties compared with surrounding genes and preserve a degree of the donor genome’s properties (Keeling and Palmer 2008). HGT is well documented in bacteria, whereas one of the most famous examples of HGT among eukaryotes is the HGT from a fungus to aphids (tribe Macrosiphini) (Moran and Jarvik 2010). The event enabled aphids to synthesize carotenoids (Moran and Jarvik 2010).

Gene duplication introduces paralogs of redundant sequence into a genome. Because they are copies, paralogs can escape the functional constraints of the original gene and undergo positive selection for new functions (Ohno 1970; Zhang 2003). A neofunctionalized gene copy will be more diverged from the originating gene compared with a copy which retains the same function (Assis and Bachtrog 2013). The relative timing of duplication events can be inferred by reconciling the gene tree with the species tree (Bansal et al. 2012; Jacox et al. 2016). A clear example of gene duplication and neofunctionalization involves the duplications of olfactory receptor genes in insects (Saad et al. 2018). Duplications created redundant paralogs and the paralogs evolved the ability to bind new ligands (Saad et al. 2018).

Although de novo gene birth, HGT and gene duplication can each give rise to new gene families, their relative influence on genomes remains enigmatic. Nonetheless, these mechanisms have clearly shaped niche transitions. For example, the plant pathogen *Pyrenophora tritici-repentis* acquired its *ToxA* virulence gene through HGT from *Stagonospora nodorum*, enabling *P. tritici-repentis* to emerge as a devastating pathogen of wheat (Friesen et al. 2006). The transition of tetrapods from water to land was mediated by the duplication and neofunctionalization of *HOX* genes, resulting in the evolution of limbs from fins (Soshnikova et al. 2013). The evidence for de novo gene birth as a driver of niche transition is indirect. One potential example involves cnidarians (*Hydra*, jellyfish, coral, etc.); several genes involved in unique predatory behaviors are cnidarian-specific, suggesting their origin is de novo (Milde et al. 2009). Although associations between new genes and niche transitions have been explored in multiple systems, few have taken a whole genome approach. Genomics may enable the discovery of all genes, including previously unknown genes, associated with a transition event.

Ectomycorrhizal (ECM) symbioses stand among the most robust examples of niche transitions in nature, having evolved independently multiple times across the fungal kingdom (Matheny et al. 2006; Tedersoo et al. 2010; Bittleston et al. 2016). ECM symbioses are mutualistic associations between fungi and plants and enable the exchange of nutrients and photosynthetically derived carbon. The associations can be identified by a morphological feature termed the Hartig net, which appears as hyphal growth between plant cortical cells (Smith and Read 2008). Research on ECM niche transitions focuses on gene loss, and gene loss appears to characterize diverse origins of the symbiosis (Kohler et al. 2015; Peter et al. 2016; Hess et al. 2018; Murat et al. 2018). Although the dynamic of gene loss may explain the repeated emergence of ECM symbiosis across distinct lineages, it does not resolve the mechanisms underpinning the evolution of the association (e.g., how ECM fungi suppress or endure plant immune responses). Gene gain is more rarely the focus of ongoing work, but gene gain may enable the formation of symbiotic structures and exchange of resources. For example, small secreted proteins (SSPs) appear to play a crucial role in fungal-plant communication and SSPs have a larger repertoire in at least some ECM species compared with asymbiotic species (Plett, Daguerre, et al. 2014; Kohler et al. 2015). Other studies have identified additional gene gains associated with the transition from a saprotrophic to ECM niche, for example expansions in cytochrome P450 and berberine bridge enzyme gene families (Hess et al. 2018). Although multiple lines of evidence suggest a role for new genes in transitions to the ECM niche, the origins of these genes remain unknown.

The fungal genus *Amanita* is an emerging evolutionary model and ideal system to test for connections between evolutionary novelty and gene gain. A single, well-resolved niche transition marks the origin of ECM *Amanita* from asymbiotic ancestral lineages (Wolfe et al. 2012; Hess and Pringle 2014; Hess et al. 2018). While genomic restructuring within ECM *Amanita* does involve the loss of plant cell wall degrading enzymes (Wolfe et al. 2012; Hess et al. 2018), the presence of gene families found only in ECM *Amanita* suggests they may also play a role in mediating the niche transition (Hess and Pringle 2014; Hess et al. 2018). By taking a closer look at these novel gene families, we aim to decipher the genetic underpinnings of the ECM symbiosis. We hypothesize novel genes enabled new functions within the emerged ECM lineage and seek to understand their sources.

Our aims are to 1) explore whether gene families unique to ECM *Amanita* function in the symbiosis and 2) identify the putative origins of these gene families. We developed a phylogenetic workflow to investigate the properties and origins of unique gene families, defined as genes only found in and shared by species of ECM *Amanita*. Analyses of transcriptomes and tests for selection support the hypothesis that unique gene families shaped the formation of the mutualism. Our workflow suggests all three gene acquisition processes were at play during the niche transition in *Amanita*, but HGT gave rise to the majority of new genes that retain enough signal for us to infer their origins.

## Materials and Methods

### Genome Sequencing and Annotation

The genomes of five *Amanita* species and one *Volvariella* species, including three ECM fungi (*A. muscaria* var. *guessowii*, *A. brunnescens*, and *A. polypyramis*) and three asymbiotic fungi (*A. inopinata*, *A. thiersii*, and *V. volvacea*), were used to identify gene families unique to symbiotic species. Genome sequencing and annotation is fully described in Hess and Pringle (2014) and Hess et al. (2018). Data of four of the genomes are available through NCBI’s GenBank (Acc. JNHV02000000, JNHW02000000, JNHY02000000, JNHZ02000000) and all data and developed bioinformatic pipelines are available at https://doi.org/10.5061/dryad.g63c748 (last accessed September 2019).

### ECM-Specific Orthologous Gene Family Calling

We first identified homologous gene families among each of the six genomes using FastOrtho implementing Markov clustering algorithm (MCL) ver. 11.294 (van Dongen 2000) and BLASTp ver. 2.7.1 (Altschul et al. 1990) with default parameters. To investigate if the inflation value parameter affected results, we ran FastOrtho five additional times with different inflation values ranging from 1.2 to 6. We defined gene families unique to ECM *Amanita* as families for which homologs are present in all three ECM *Amanita* but not in any of the three asymbiotic fungi. A similar approach was used in Hess et al. (2018), but the resulting estimates are different from ours because different parameters were used.

### Identifying Selection Pressures on Gene Families Unique to ECM *Amanita*

We next sought to understand which gene families possess signals of purifying selection (d*N*/d*S* < 1). The putative protein sequences of all genes from each gene family were first aligned with MAFFT ver. 7.149b (Katoh et al. 2002) and then trimmed with trimAl ver. 1.4.rev15 (Capella-Gutiérrez et al. 2009) using default parameters. A phylogeny for each gene family was built with RAxML ver. 7.2.8 (Stamatakis 2006), using the trimmed protein alignment, and applying gamma rate heterogeneity and the best substitution model (either JTT, LG, or WAG) as determined by AICc values calculated by ProtTest ver. 3.4 (Darriba et al. 2011). The DNA sequences of coding regions (CDS) in each gene family were also aligned based on a codon substitution matrix, using PRANK ver. 140603 (Loytynoja and Goldman 2005). Protein phylogenies and CDS alignments were used to test the alternative hypothesis of d*N*/d*S* bias away from neutral selection using the codeml program implemented in PAML ver. 4.8 (model = 0, CodonFreq = 3, fix_kappa = 0, fix_omega = 0 vs. 1) (Yang 2007).

### The Differential Expression of Gene Families Unique to ECM *Amanita*

To test if one of the ECM *Amanita*, *A. muscaria*, preferentially expresses genes unique to ECM species during symbiosis, we compared expression patterns of genes in each of three categories: 1) genes conserved across the six species (*n* = 5,264), 2) ECM-*Amanita*-unique genes (*n* = 272), and 3) orphan genes (*n* = 4,989) (genes only found in *A. muscaria* var. *guessowii*). To assess if the proportion of genes upregulated in symbiosis (=the number of genes upregulated in symbiosis/total number of genes) is higher for ECM-*Amanita*-unique genes compared with conserved or orphan genes, we first identified all genes upregulated in symbiotic cultures. We retrieved the expression read count table generated from both symbiotic root tips and axenic cultures of *A. muscaria* var. *muscaria* from the JGI genome portal (project ID: 1025043) (Kohler et al. 2015). Because the expression data (used for training JGI’s genome annotation and generating expression table) are taken from *A. muscaria* var. *muscaria* but the genome assembly was generated from *A. muscaria* var. *guessowii*, we mapped our genome annotation (trained with transcriptome from *A. muscaria* var. *guessowii*) to the expression data by finding the best hit of each gene in our genome annotation to the gene sequences used in the expression data with BLASTp (*E* value = E−3). A Wald test was performed to screen for differentially expressed genes using the R package DESeq2 (Love et al. 2014). Upregulated genes were defined using FDR adjusted *P* values < 0.01 and an expression level fold-change > 2, 4, or 8. The proportion of upregulated genes was compared across the three categories of gene families using Fisher’s exact test and an FDR correction for the *P* values. We also performed the same analyses to assess if the genes belonging to any of the three categories mentioned above are upregulated in axenic culture.

### Identifying Origins of Gene Families Unique to ECM *Amanita*

#### Overview

Before describing our workflow in greater detail in the sections below, we outline our basic approach: In each family unique to ECM *Amanita*, we selected the longest gene from the *A. muscaria* var. *guessowii* genome as a query to find homologous genes in an in-house, curated proteome database designed to represent the diversity of the three domains of life (see below and supplementary files 1 and 2, Supplementary Material online) (Staehlin et al. 2016), using BLAST. We considered genes with no hits as candidates for de novo gene birth. Next, to identify HGT events from the remaining genes, we compared gene trees to the species tree to look for incompatibilities. If a gene was found in a monophyletic clade without *Amanita* species, the gene was considered as a candidate for HGT. After excluding candidates for de novo gene birth and HGT, we identified potentially duplicated genes by looking for orthologs in asymbiotic species and ECM-paralogs in ECM *Amanita* (we define ECM-paralogs as the paralogs derived from duplications coinciding with the niche transition). If both orthologs and ECM-paralogs were found, the gene was considered as a candidate for duplication. Finally, we attributed genes whose homologs are only absent from asymbiotic *Amanita* and *Volvariella* to the phenomenon of selective retention (multiple independent deletion events in the asymbiotic lineages but not in ECM *Amanita*).

#### De Novo Gene Birth

To identify putative homologs of the gene families unique to ECM *Amanita*, we curated an in-house genome database with 354 fungal (supplementary file 1, Supplementary Material online; last accessed May 2015), 1,153 prokaryotic (Staehlin et al. 2016), and 88 plant genomes (supplementary file 2, Supplementary Material online; last accessed October 2017). Then, we compared the protein sequence representing the longest *A. muscaria* var. *guessowii* gene of each ECM unique gene family to the database using uBLAST implemented in uSearch ver. 8.0.1517 (Edgar 2010). To maximize the probability of finding potentially homologous sequences, we screened using a conservative *E* value of E-3 and minimum identity of 0.25. If matches did not return sequences of all three ECM *Amanita* for a given gene probe, results were considered inconclusive and these genes were discarded from the analyses entirely. When results consisted of only the three ECM species, and no other hits, gene families remained in consideration as possible de novo gene families. The probe sequences were aligned to the NCBI GenBank (https://www.ncbi.nlm.nih.gov/genbank/; last accessed October 2019) and UniProt (http://www.uniprot.org/; last accessed October 2019) databases using BLASTp with the same *E* value cutoff of E−3 and an additional filter for low complexity regions to check for homologs in either database. Sequences without matches were considered as candidates for de novo gene birth.

Next, to explore whether identified gene families are potentially derived from noncoding sequence, we identified the syntenic block of each target gene by matching five upstream and five downstream genes across the three ECM genomes. The same upstream and downstream genes were identified in the three asymbiotic genomes to locate syntenic blocks and putatively homologous, noncoding sequences. The putatively homologous sequences were aligned to candidate de novo genes to explore synteny with MAFFT. We further used HISAT2 Galaxy Version 2.1.0 (Kim et al. 2015) with default parameters to map the Illumina transcriptomic raw reads sequenced from mycelia of asymbiotic species cultured in litter (*Amanita*) or potato dextrose broth (*V. volvacea*) (Acc. SRR089758, SRR619832, SRR7694628) (Bao et al. 2013; Hess et al. 2018) onto the three asymbiotic reference genomes to understand if the homologous regions of de novo genes in asymbiotic lineages are expressed.

#### Horizontal Gene Transfer

After excluding gene families categorized as stemming from de novo gene birth as well as gene families for which no strong conclusion could be made, we sought to identify gene families derived from HGT events using the putative homologs in our curated genome database. We first reconstructed a crude phylogeny for each gene to identify potential HGT events. We took uBLAST results and used OrthoMCL ver. 1.4 (Li et al. 2003) with an inflation value of 1.5 to cluster the uBLAST results returned for each gene and identify the genes in the same cluster with any target gene, eliminating highly diverged sequences. Refined uBLAST results were aligned with MAFFT and trimmed with trimAl using default parameters. (TrimAl failed to trim Family 12,764, resulting an empty alignment, and so this family was not considered further). Preliminary phylogenies of the aligned and trimmed protein sequences were then constructed using FastTree ver. 2.1.7 (Price et al. 2010) and compared with the fungal taxonomy (Spatafora et al. 2017): genes of putative HGT families should nest within clades unrelated to *Amanita* (e.g., within the Ascomycota).

We next generated more accurate gene phylogenies for downstream analyses only with those gene families tentatively identified as resulting from HGT. Phylogenies were generated using a subset of sequences: for each putative HGT event, we identified branches with bootstrap support of >90% housing between 100 and 350 sequences and including at least one gene from each of the three ECM *Amanita* species. These data sets were aligned and trimmed again and used to generate new trees using RAxML (Stamatakis 2006) with best evolutionary models identified by ProtTest based on AICc values.

To rigorously reject the null hypothesis of vertical inheritance of genes, we compared our unconstrained trees with vertical-inheritance-constrained trees by using AU tests to identify the best phylogenetic model for each putative HGT family (Shimodaira 2002). We manually constructed the constraint trees by enforcing the null hypotheses of vertical inheritance (either by enforcing monophyletic Agaricales including *Amanita*, or monophyletic putative donor group) in Mesquite ver. 3.2 (http://mesquiteproject.org; last accessed May 2017) (supplementary file 3, Supplementary Material online). Each constraint tree was used to reconstruct a RAxML phylogeny. To test if the unconstrained phylogenies (suggesting HGT) were strongly favored over constrained phylogenies (indicative of divergence in accordance with speciation), we used the per-site likelihood values of both the original unconstrained and constrained phylogenies to perform AU tests implemented using CONSEL ver. 1.2 (Shimodaira and Hasegawa 2001).

Finally, we explored the gene structure (the intron sites) of genes associated with putative HGT events. We hypothesize the structures of putative HGT genes will be more similar to the genes from putative donors than to genes from Agaricales. To compare putative HGT genes with homologous genes from putative donors and homologous genes from Agaricales, we used GenePainter (Hammesfahr et al. 2013) to visualize YAML-formatted gene structures generated with Webscipio (Hatje et al. 2011) and protein alignments from MUSCLE ver. 3.8.31 (Edgar 2004).

#### Gene Duplication

We next tested whether remaining gene families (gene families not resulting from either de novo gene birth or HGT) might result from duplication and the subsequent rapid evolution of paralogs. We took a two-step approach: first, we looked for orthologs of putative rapidly evolving genes in asymbiotic species; next, we identified ECM-paralogs (paralogs derived from gene duplications coinciding with the niche transition). The closest asymbiotic ortholog to the new gene family served as an outgroup, allowing us to distinguish new gene families from ECM-paralogs among ECM species.

To identify orthologs in asymbiotic species, we generated a more robust phylogeny using RAxML with the uBLAST results generated for the HGT analysis. If the MCL-reduced data set housed more than 800 sequences, the data set was further trimmed using the FastTree trimming method described above; if the MCL-reduced data set contained 30–800 sequences, the MCL-reduced data set was used; if the MCL-reduced data set consisted of fewer than 30 sequences, the entire data set was used to reconstruct the phylogeny. To generate phylogenies we used MAFFT, trimAl, ProtTest, and RAxML as above. Next, to identify potential orthologs in asymbiotic species, we first rooted each phylogeny using its midpoint and then split each phylogeny (of each gene family) into single gene trees with TreeKO algorithm implemented in etetoolkit (Huerta-Cepas et al. 2010; Marcet-Houben and Gabaldón 2011). The TreeKO algorithm splits the phylogenies into multiple single gene trees by trimming off branches that represent duplication events until every species only has a single gene in any single gene tree, and therefore genes in each single gene tree can be treated as orthologs (Marcet-Houben and Gabaldón 2011). We analyzed single gene trees to test if each tree houses 1) only sequences from ECM species or 2) sequences from both ECM and asymbiotic species. Scenario 1) would suggest no orthologous genes can be found in asymbiotic species so selective retention in ECM species (multiple deletions in asymbiotic species) is the more parsimonious explanation of why these families being identified as families unique to ECM *Amanita*. Scenario 2) suggests the presence of orthologs in asymbiotic species.

Finally, to detect clear signals of gene duplications originating with the niche transition, we sought to identify ECM-paralogs associated with the transition to ECM niche. We first identified all nodes between 1) the most recent common ancestor (MRCA) of a given family (determined by FastOrtho), and 2) the MRCA of this family and the most phylogenetically proximate asymbiotic ortholog(s). We then identified ECM *Amanita* homologs diverging from the abovementioned intermediary nodes, and labeled these homologs as ECM-paralogs. If ECM-paralogs are present and no homolog from other species clusters with ECM-paralogs, we consider the origin of the family to be gene duplication and look for a bootstrap value ≥80 supporting the ECM-paralogs.

To test if the duplicated genes experienced novel selection pressure, we trimmed off the tips that are not from *Amanita* and *Volvariella* in the phylogeny of each gene family and labeled the branch of duplication as “foreground.” The phylogeny and their CDS alignment from PRANK was then analyzed by aBSREL (Smith et al*.* 2015), implemented in HYPHY ver. 2.5.1 (Kosakovsky Pond et al. 2005), to test if the duplication branch (foreground branch) has a proportion of codons that has experienced positive selection (d*N*/d*S* > 1).

#### Selective Retention in Asymbiotic *Amanita* and *Volvariella*

Gene families not fitting into any criterion described above were considered as the results of selective retention (multiple deletions in asymbiotic species). To be stringently considered as a gene family that has undergone selective retention, uBLAST results of genes within a given family must not contain any of the three asymbiotic species.

## Results

### Number and Properties of Unique Gene Families

The number of gene families found only in the three ECM *Amanita* genomes ranged from 89 to 120. Changing the parameter settings for FastOrtho greatly impacted the number of gene families identified as unique. The total number of gene clusters increased from 8,694 to 11,436 as the inflation value increased (supplementary file 4, Supplementary Material online). Because we have no prior knowledge of gene function we decided to use the default inflation value of 1.5, which balances sensitivity and selectivity and fits enzyme family nomenclature according to which reaction an enzyme catalyzes (EC annotation) (Li et al. 2003).

**Fig. 2.**
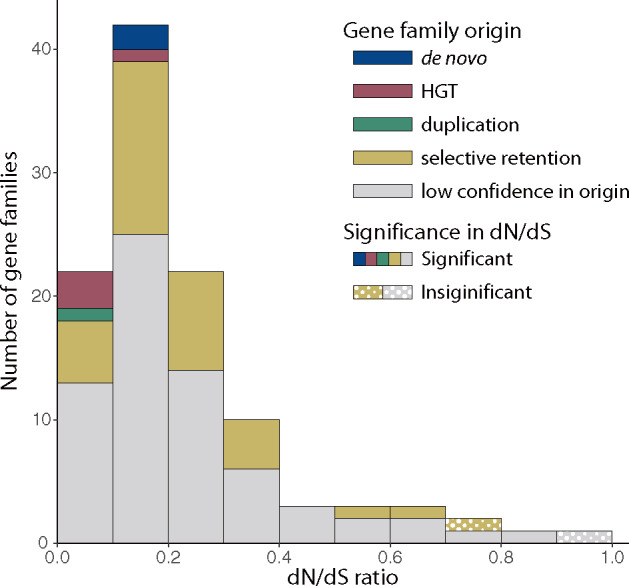
Frequency distribution of the d*N*/d*S* ratios of the 109 gene families unique to ECM *Amanita*. Most gene families (98.2%) have experienced purifying selection with d*N*/d*S* ratios significantly lower than one (*P* value < 0.05).

Using this inflation value we identified a total of 9,429 gene families and identified 109 gene families as unique to the three ECM *Amanita* genomes (supplementary file 5, Supplementary Material online). Among the 109 gene families, 107 are undergoing significant purifying selection (d*N*/d*S* < 1; LRT *P* value <0.05). Of the gene families experiencing purifying selection, values of d*N*/d*S* range from 0.00256 to 0.8504 (fig. 2). The d*N*/d*S* ratio provides evidence that genes from these gene families encode proteins and are not annotation artifacts. Two gene families had d*N*/d*S* ratios close to one suggesting either the genes do not code for functional proteins or the genes are under neutral selection (in other words, natural selection does not influence the evolutionary trajectory of these genes).

**Fig. 3.**
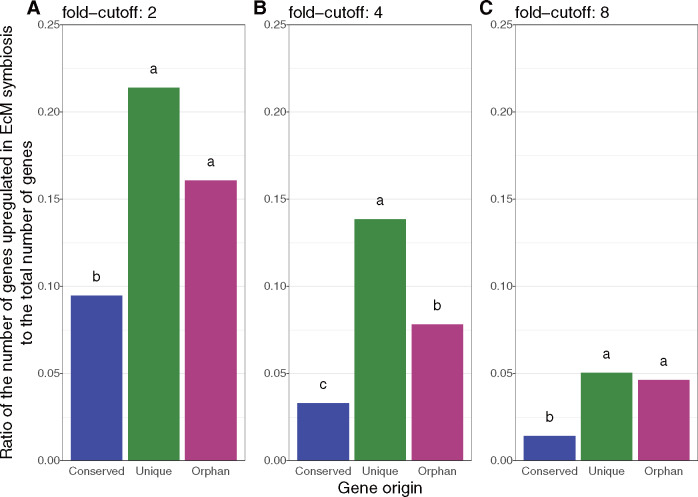
The ratio of the number of genes upregulated in ECM symbiosis to the total number of genes for conserved (*n* = 5,264), unique (*n* = 272), or orphan families (*n* = 4,989). Different fold cutoffs (two, four, and eight) are used to define upregulation in subplots *A*, *B*, and *C*. Fisher exact test *P* value < 10^−4^ for all cutoffs. Letters mark significant (adjusted *P* value < 0.05).

When using a fold-change cutoff of four, a significantly higher proportion of genes in the 109 unique gene families were upregulated in ECM root tips, compared with orphan genes found only in *A. muscaria* and genes conserved across all six species. In addition, a significantly higher proportion of orphan genes were upregulated in ECM root tips, compared with genes in conserved gene families (fig. 3). However, the difference between unique and orphan genes was not significant using a fold-change cutoff of two or eight (fig. 3*A* and *C*). The difference between unique and conserved genes was significant regardless of the fold-change cutoff. In axenic cultures, unique and orphan were upregulated compared with conserved families when any fold-change cutoff was applied (supplementary file 6, Supplementary Material online).

### De Novo Gene Birth

Based on uBLAST searches of our curated genome database, six families were identified as candidates for de novo gene birth. However, additional BLAST searches in the GenBank and UniProt databases detected putative homologs for four of these families, leaving only two gene families as candidates for de novo gene birth (families 1,476 and 3,446).

For each of the two gene families, each ECM *Amanita* species has only a single gene copy. The two putative de novo gene families have d*N*/d*S* ratios significantly lower than one (family 1,476: 0.174 and family 3,446: 0.198; codeml LRT *P* value <10^−20^), suggesting the genes are experiencing strong natural selection. We returned to the transcriptomic data to probe expression patterns of the genes from these two gene families. The gene representing gene family 1,476 is expressed constitutively in both symbiotic and axenic cultures (14–26 RPKM in each treatment; RPKM=reads per kilobase of exon model per million mapped reads). Transcripts of the gene representing the gene family 3,446 are detected but are not present at levels greater than 1 RPKM in any treatment. Although the evidence suggests these are real genes, there is no evidence for the upregulation of either of the two gene families in ECM root tips.

Neither of the two genes has a known function. The lengths of the proteins are 178 (family 1,476) and 297 (family 3,446) amino acids. Genes from the two gene families have a GC content of 49.9% and 52.0%, respectively, and these GC contents more closely resemble the CDS of conserved genes (49.4%) compared with intergenic regions (46.0%) although each is presumably derived from an intergenic region (supplementary file 7, Supplementary Material online). In addition, each gene possesses at least one intron (gene 1,476 from *A. muscaria* var. *guessowii* has two introns, but genes in the same family from *A. brunnescens* and *A. polypyramis* have only one intron). Introns are less commonly reported in genes derived from de novo gene birth, but there may be a bias because most research focuses on recently birthed genes. Our data suggest the evolutionary history of ECM *Amanita* is long enough that these two gene families acquired introns.

**Fig. 4.**
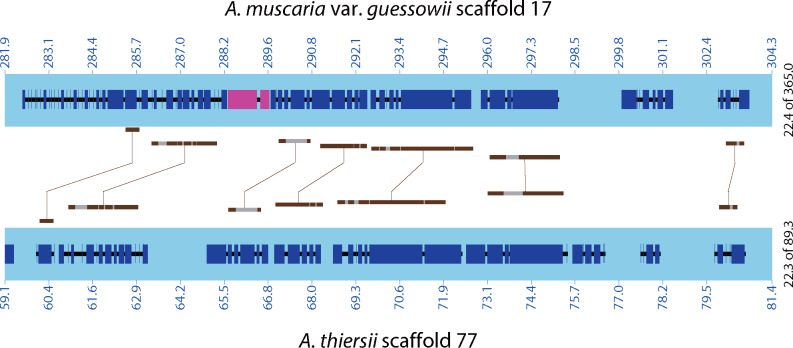
Syntenic structure between scaffold 17 of *A. muscaria* var. *guessowii* (ECM) and the scaffold 77 of *A. thiersii* (asymbiotic) highlighting the location of one gene (AmumRNA.3446.1; purple) putatively identified as deriving from de novo gene birth. Light blue, scaffolds; Dark blue, exons; Black, introns; Brown, homologous genes (determined by BlastX); Gray, nonalignable regions. Units: kbp.

Gene family 1,476 is located within a nonsyntenic region. We hypothesize that this gene family is located in a relatively variable region. In contrast, genes from family 3,446 are located within a conserved region across the three species (fig. 4). We attempted to find the homologous noncoding sequence of these genes by aligning homologous regions from ECM and asymbiotic species. However, the pairwise identities (the proportion of aligned nucleotides) of these multiple sequence alignments were low and ranged from 42.0% to 53.3%. When we searched for evidence of expression of the homologous sequences in asymbiotic species, we found a few raw reads of this region in the transcriptome of all three asymbiotic species: *A. inopinata* (1 read), *A. thiersii* (3 reads) and *V. volvacea* (9 reads) (supplementary file 8, Supplementary Material online). The low abundance of transcripts from this region leaves open the question of whether this homologous, presumably noncoding region is actually transcribed by the fungus in nature. Although no gene is present in these regions in *A. inopinata* and *A. thiersii* according to their annotations, three genes are annotated in the homologous region from *V. volvacea*, and one gene is responsible for its transcriptomic reads. However, these three genes show no homology with gene family 3,446.

### Horizontal Gene Transfer

**Fig. 5.**
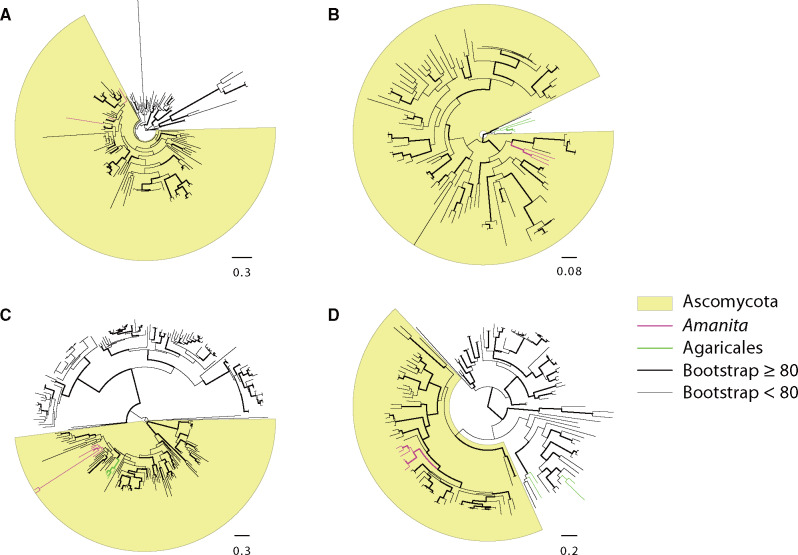
Protein trees of the four putative HGT gene families. (*A*) family 2,813 (with unknown function); (*B*) family 10,418 (ACC deaminase); (*C*) family 11,987 (metal binding acid phosphatase); (*D*) family 12,806 (with unknown function).

**Fig. 6.**
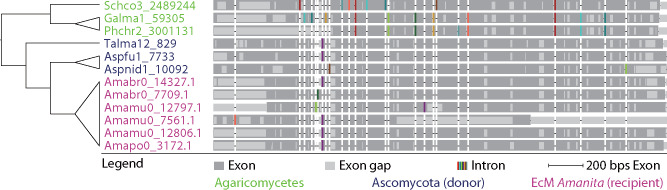
Gene structures of the HGT family 12,806 (with unknown function). The intron structures of ECM *Amanita* genes deriving from HGT are more similar to their putative donors’ homologs than to homologs belonging to other Agaricales members.

Comparing the species tree and crude gene phylogenies generated by FastTree, six gene families emerged as candidates for HGT. After building the RAxML gene phylogenies, one gene family was no longer placed with the putative donor lineage and therefore we did not consider it further. Of the remaining five families, AU tests failed to reject vertical inheritance as a possibility for one of the gene families (7,854). In families 11,987 and 12,806, AU tests rejected the two hypotheses which would suggest vertical inheritance: 1) all genes from Agaricales (including *Amanita*) forming a monophyletic group (*P* values < 0.05) and 2) the genes from the putative donor forming a monophyletic group (*P* values < 0.01); these tests strongly suggest this family derived from HGT. In two other families, only one hypothesis suggesting vertical inheritance was rejected. In family 10,418, only the first hypothesis was rejected (*P* value = 0.001), whereas in family 2,813, the second hypothesis was rejected (*P* value = 0.01). However, we were unable to test for the first hypothesis in family 2,813 (no other Agaricales homologs were found) (fig. 5; supplementary file 9, Supplementary Material online). In summary, we consider each of these four families (2,813, 10,418, 11,987, and 12,806) to be the result of HGT. Gene structures provide additional evidence for the HGT of gene family 12,806. In this family the ECM *Amanita* genes share more intron sites with homologs from putative donors than with homologs from other Agaricales species (fig. 6).

ECM *Amanita* genes in three of the four HGT families form monophyletic groups in the donor lineage. We hypothesize the genes in family 2,813 do not form a monophyletic group because an insufficient phylogenetic signal leads to poorly resolved branches: bootstrap values supporting polyphyly range from 3 to 36. Each of the four families clusters inside Eurotiomycetes, or inside Ascomycota but with Eurotiomycetes as the sister group to the HGT genes. However, there is no evidence that the HGT genes are linked in either the donor lineage or *Amanita* genomes so there is no support for a single transfer of the four genes (e.g., as a gene cluster), which would be more parsimonious.

GO terms were assigned to three HGT families, including GO:0008660 [1-aminocyclopropane-1-carboxylate {ACC} deaminase activity], GO:0009310 (amine catabolic process), GO:0030170 (pyridoxal phosphate binding), GO:0003993 (acid monophosphatase activity), GO:0046872 (metal ion binding), GO:0016787 (hydrolase activity), GO:0016788 (esterase activity) (supplementary file 5, Supplementary Material online). All HGT gene families have a d*N*/d*S* < 1 (0.066–0.178) and are expressed. Only the gene from family 10,418 (an ACC deaminase) is upregulated in symbiotic cultures, and it is 52-fold overexpressed.

### Gene Duplications

**Fig. 7.**
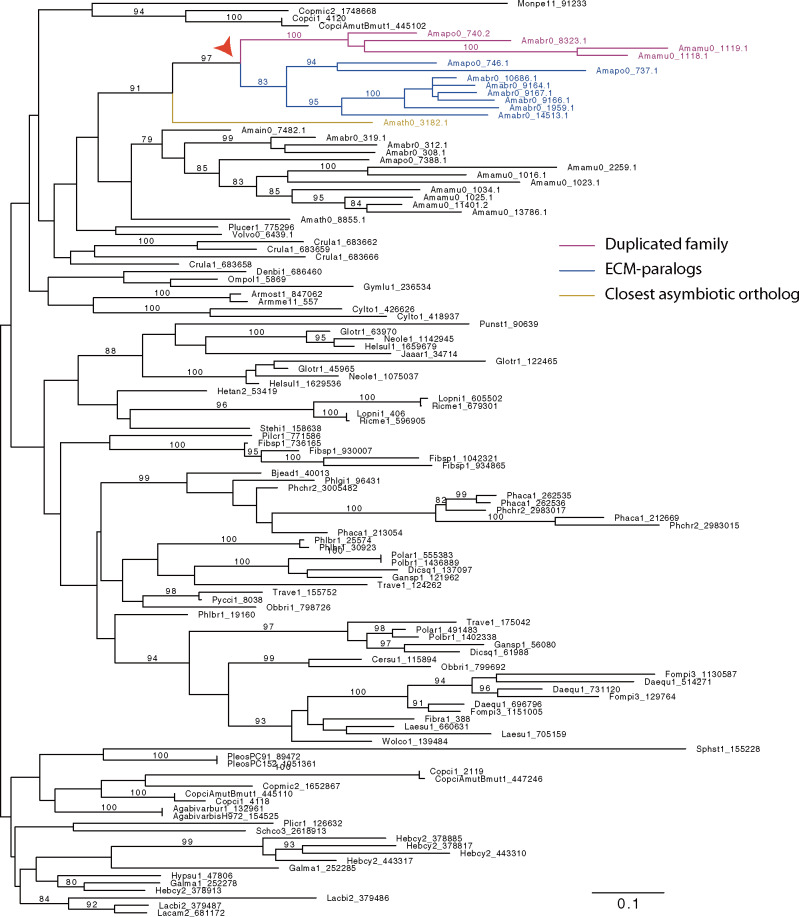
Phylogeny of gene duplication in family 1,119 (DUF6). Branches with bootstrap values ≥80 are labeled. Arrow head: duplication generating family 1,119.

Orthologs of 16 remaining gene families were found in asymbiotic species, and clear evidence of ECM-paralogs is found for four of these 16 families. However, common ancestry with ECM-paralogs is supported by a bootstrap value ≥80 for only one family, family 1,119 (fig. 7). But only two species (*A. brunnescens* and *A. polypyramis*) possess these ECM-paralogs, in *A. muscaria* var. *guessowii* there are no family 1,119 ECM-paralogs. The closest asymbiotic ortholog of this family is clustered with some but not all of the ECM-paralogs by FastOrtho, which suggests a divergence after duplication. We hypothesized that this family experienced positive selection shortly after gene duplication. However, we failed to reject the null hypothesis of an absence of positive selection (aBSREL LRT *P* value = 0.063). In addition, these genes are not located in syntenic regions and so we are unable to test if family 1,119 is the more recent ECM-paralog.

This family is transcribed and annotated as a membrane protein (GO:0016020) (supplementary file 5, Supplementary Material online). In the transcribed product, no signal peptide was predicted by SignalP ver. 5.0 (Almagro Armenteros et al. 2019), 10 transmembrane domains were found by TMHMM ver. 2.0 (Krogh et al. 2001), and the product was predicted to be located on Golgi apparatus membranes by DeepLoc ver. 1 (Almagro Armenteros et al. 2017). HMMER 3.3 (Potter et al. 2018) predicted the family belongs to the DUF6 (domain of unknown function 6 or EamA) family. The limited evidence of positive selection and the ability to differentiate the functions of the newly duplicated genes and their paralogs prevents any inference of neofunctionalization following duplication for this family.

### Selective Retention

Thirty-four gene families unique to ECM *Amanita* appear to be the result of selective retention (equivalent to multiple deletions in asymbiotic lineages). Based on uBLAST and an *E* value < E−3, these gene families lack homologs in the three asymbiotic species. False positive signals of selective retention may result from either our choice of *E* value cutoff, HGT from unconsidered lineages, or false negatives in annotation. However, our estimate remains conservative because some selectively retained gene families may house paralogs in asymbiotic species that diverged before the origin of the ECM symbiosis, and these families are not considered here.

## Discussion

### The Origins of Gene Families Found Only in Symbiotic *Amanita*

New gene families shape genome evolution and can drive adaptation to novel niches (Friesen et al. 2006; Milde et al. 2009). Among ECM *Amanita*, the genes of unique gene families (gene families found in the three ECM species but not found in closely related asymbiotic fungi) are upregulated in ECM root tips compared with the genes of conserved or orphan gene families, suggesting the new gene families acquired during the niche transition function during symbiosis. Most of the genes of unique gene families have also undergone strong natural selection.

We discovered evidence for the precise origins of 41 families. Thirty-four families are inferred as unique because of selective retention, and the other seven appear as truly new gene families, derived from either de novo gene birth (two), HGT (four), or divergence after gene duplication (one). The low number of de novo gene families is not surprising. The high turnover rate of de novo genes in any genome results in a low preservation of de novo families (Palmieri et al. 2014). Although the mechanism(s) driving HGT in fungi remain elusive, accumulating evidence suggests HGT is a key to evolutionary innovation in the fungal kingdom (Soanes and Richards 2014). Finding four gene families derived from HGT suggests HGT was also critical to the changes in *Amanita* ecology, but this discovery might also reflect the better preservation of the phylogenetic signal of HGT over time, compared with signals from de novo gene birth or gene duplication. Unexpectedly, using our workflow we only identified one new gene family derived from gene duplication, contrary to expectations that gene duplication is a common source of new genes in fungi and other eukaryotes (Ohno 1970; Zhang 2003; Wisecaver et al. 2014). The discrepancy may result from the use of a MCL to identify new families, as the algorithm can consolidate paralogs into a single gene family if they have not undergone rapid divergence (Li et al. 2003), but the discrepancy may also result from a lack of phylogenetic support for the monophyly of paralogs. Changing the parameters used with MCL may influence how clusters are identified (Li et al. 2003). For example, whereas we identified 109 families as unique to ECM genomes Hess et al. (2018) identified 171 unique gene families. We used no match cutoff or identity cutoff and used an inflation value of 1.5, whereas Hess et al*.* (2018) used a match cutoff of 60%, an identity cutoff of 30%, and an inflation value of 3. Parameter choice involves a balance: choosing more stringent clustering results in greater numbers of clusters (Hess et al. 2018) and may lead to the identification of greater numbers of duplicated gene families. However, whether these gene families are functionally diverged enough to be judged as novel is an open question, and choosing higher cluster tightness can break orthologs into different families (Li et al. 2003). In summary, the number of gene origins inferred reflects the number of gene family birth events, rates of gene turnover, the decay of phylogenetic signal and the choice of clustering algorithm parameters.

Research on the origins of new genes has focused on orphan genes found in single species and not gene families found across closely related species. Most of these orphan genes are more recent than the niche transition in *Amanita* [the origin of symbiosis among *Amanita* fungi dates to 80 million years ago {Varga et al. 2019}]. By focusing on younger genes, these studies take advantage of opportunities to trace homologies and syntenies to elucidate the molecular mechanisms of the emergence of new genes (Donoghue et al. 2011; Arendsee et al. 2019). Moreover, HGT and duplication events are generally left out of their foci.

Other studies on gene family origins have used two major approaches to identify different evolutionary events. The first approach estimates the gene turnover rate with the gene counts of a family in different species, typically using gain-and-death or birth-death-and-innovation (BDI) models (Librado et al*.* 2012). The second approach uses a species tree/gene tree reconciliation method to identify duplication, gene loss and HGT (the DTL scenario) (Bansal et al. 2012; Jacox et al. 2016); other algorithms also incorporate incomplete lineage sorting (DTLI) (Stolzer et al. 2012). Because innovation events in the BDI model account for both de novo gene birth and HGT (Karev et al. 2002), this first approach does not provide information on the different mechanisms mediating the origins of new gene families. However, the second approach does not account for de novo gene birth (Tofigh et al. 2011). In addition, methods using the DTL scenario require a well-curated species tree for not only the species of interest but also the putative donors for HGT (Bansal et al. 2012; Jacox et al. 2016) and these are not always available. To account for de novo gene birth, HGT and gene duplication simultaneously, and to avoid a reliance on a well-curated species tree, our workflow includes 1) gene clustering to identify new gene families, 2) the search for homologs in a curated genome database, 3) phylogenetic analyses designed for different origin hypotheses, followed by 4) integration of additional support from analyses of for example, gene structure, composition, and synteny (Gluck-Thaler and Slot 2015).

### De Novo Gene Families Have Lost Some Properties of De Novo Genes

Interest in de novo gene birth is growing. However, misidentification of de novo genes can result from either the rapid evolution of an extant gene or a poorly resolved comparison between a putative de novo gene and an incomplete genome database (Moyers and Zhang 2015). Casola (2018) suggests investigating four gene features to avoid misidentification and enable recognition of real de novo gene families: the absence of homologs in other taxa, a lack of conserved domains, conserved synteny, and substitutions enabling genes encoding proteins (e.g., generating a start codon). We successfully identified the first three features for at least one family (3,446). We were not able to detect the last feature (substitutions enabling genes encoding proteins) because of the dissimilarity between the putative de novo genes and their homologous noncoding sequences. Because de novo gene families can evolve from sequences encoding long noncoding RNAs (Schlötterer 2015), we searched for transcripts of noncoding sequences orthologous to putative de novo gene families in asymbiotic species. However, the low number of raw reads we discovered prevents firm conclusions as to whether or not these regions are transcribed by the fungi in nature.

The genes of de novo gene families are reported to possess several distinctive characteristics, including short gene lengths (around 300–400 bp) and few to no introns (Wu and Knudson 2018). Sequences may be GC poor or have GC content similar to conserved genes, depending on the species (Palmieri et al. 2014; Wu and Knudson 2018). However, the two de novo gene families we identified have a structure and composition consistent with the conserved gene families in *Amanita* genomes. Combined with the absence of similarities between de novo gene families and their homologous noncoding sequences, these observations lead us to conclude that these gene families have had sufficient time to become ameliorated and now resemble other coding genes (Marri and Golding 2008).

De novo gene families of animals and plants have low gene expression levels and higher tissue-specific expression compared with older genes (Schlötterer 2015), especially genes expressed in animal testes (Begun et al. 2007). One of the two de novo families we identified has a higher expression rate in axenic mycelium compared with ECM root tips whereas the other is not differentially expressed so these genes are unusual compared with the genes of the other 109 gene families. We hypothesize that these gene families are not directly involved in the plant-fungal interaction, but with other processes shared among ECM *Amanita* species.

### Genes Horizontally Transferred to ECM *Amanitas* Are from Ascomycota, and We Hypothesize ACC Deaminase (Family 10,418) Was Directly Involved in the Transition to Symbiosis in *Amanita*

As with identification of de novo gene birth, strategies to identify HGT using only similarity as a criterion are also problematic (Guindon and Perrière 2001). Keeling and Palmer (2008) have suggested gene-species phylogenetic incongruence as the gold standard for HGT detection. Based on that gold standard, we identified four unique gene families originating from HGT. In two families, we successfully rejected the null hypotheses that 1) genes from Agaricales and *Amanita* are monophyletic and 2) the genes from donor group itself are monophyletic, but we failed to reject one of the null hypotheses for the other two families. The failure to reject all null hypotheses may be caused by accumulated substitutions in transferred genes, or too few taxa in our curated genome database. In addition, one gene family showed a high similarity of exon/intron structure to its putative donor, providing further evidence for HGT. These four gene families are not the first record of HGT to ECM *Amanita*. We previously reported that genes of carbohydrate esterases family 1 (CE1s) were transferred to ECM *Amanita* from bacteria (Chaib De Mares et al. 2015), but these genes are not found in *A. polypyramis* and thus do not fit our current definition of gene families unique to ECM *Amanita*.

Interestingly, all four HGT families are inferred to have been transferred from Ascomycota and specifically from Eurotiomycetes. Multiple HGTs to a single lineage from the same donor have been reported before and are usually enabled by their transfer as a gene cluster or syntenic block (Slot 2017), but “highways” of HGT between lineages have also been inferred (Qiu et al. 2016). In the case of HGT to ECM *Amanita*, no evidence of physical linkage in either *Amanita* or the putative donors was found. The lack of synteny suggests either these genes were transferred independently or the genes migrated into different genomic locations after HGT.

HGT genes may facilitate adaptation to new niches among the fungi (Soanes and Richards 2014). Of the four gene families transferred to ECM *Amanita*, we consider the family encoding ACC deaminase as the best candidate for driving niche transition because of its expression patterns and putative function. The gene from this family is upregulated in ECM root tips more than 52-fold, which is the highest fold difference among all genes from the 109 gene families. ACC deaminase can remove the amine group from an ACC and produce α-ketobutyrate (Honma and Smmomura 1978). ACC is the immediate precursor of ethylene, and ACC deaminase can therefore inhibit the ethylene signaling pathway, a negative regulatory pathway of ECM symbioses (Plett, Khachane, et al. 2014). In fact, the ACC deaminase knockouts of bacteria involved in a similar mutualistic system, nitrogen fixing rhizobia, are less capable of nodulation compared with the wild types (Nascimento et al. 2016). In addition, in arbuscular mycorrhizal fungi, the *SP7* gene also inhibits the ethylene signaling pathway (Kloppholz et al. 2011). We hypothesize the ACC deaminases of ECM *Amanita* reduce the concentration of ACC and ethylene in ECM roots during symbiosis, and the reduction of ethylene in ECM root tips enables the lateral branching of ECM roots and formation of the Hartig net. Moreover, our finding may provide new evidence of a molecular convergence among mycorrhizal and root-nodulating associations. Lastly, we note ACC deaminase genes are also found in other ECM Amanita genomes not included in our analyses, *A. bisporigera*, *A. phalloides*, *A. jacksonii*, etc. (van der Nest et al. 2014; Pulman et al*.* 2016).

### No Significant Evidence for Positive Selection on Newly Duplicated Genes

Many established algorithms are available to detect gene duplication. However, because we are specifically interested in potential duplication events coinciding with niche transition, we chose to detect the orthologs and ECM-paralogs of each of our identified unique gene families. Using this strategy, the only family duplicated during niche transition has a closest ortholog in *A. thiersii* and ECM-paralogs in *A. brunnescens* and *A. polypyramis*. Multiple evolutionary scenarios can explain the phylogenetic topology (e.g., duplication before the MRCA of *A. inopinata* and ECM *Amanita*, followed by deletion of both homologs in *A. inopinata*), but a single deletion in *A. inopinata* and duplication along the branch leading to niche transition is the most parsimonious explanation. We are also able to detect homologs in *A. inopinata* and *V. volvacea*, but these homologs are from diverged phylogenetic clusters, suggesting the family possesses a dynamic evolutionary background.

Gene duplication provides functional redundancy and paralogs often experience novel selection pressure, undergoing neofunctionalization (Saad et al. 2018). An extreme case suggests asymmetric evolutionary rates between two paralogs (or ohnologs to be precise) in yeast (Byrne and Wolfe 2007). However, in the new gene family derived from duplication in ECM *Amanita*, we failed to detect signals of positive selection along the branch leading to the new gene family cluster. We hypothesize synonymous substitutions have reached saturation at selected sites and the substitutions have removed the trace of positive selection (Gharib and Robinson-Rechavi 2013). However, it is also possible the family emerged as a result of nonselective events.

The gene family stemming from a recent duplication encodes two DUF6 domains in the form of a 5 + 5 transmembrane protein (each “5” is one DUF6 domain). Many proteins with this configuration are transporters, for example, O-acetylserine/cysteine export proteins and nucleotide sugar transporters (Västermark et al. 2011), but at least one gene, *PecM*, is involved in the degradation of pectin and cellulose (Rouanet and Nasser 2001). Although this gene family was identified as a new family, conserved in ECM *Amanita*, there is no evidence for its differential expression. We hypothesize that if this family is not solely the result of stochastic evolution, this gene family could be involved in transmembrane transport, substrate degradation by absorptive hyphae or controlled post-transcriptionally.

## Conclusion

We developed a workflow for identifying the origins of new gene families unique to symbiotic fungi and not found in closely related free-living fungi. Among the 109 new gene families present in ECM *Amanita*, two, four and one families appear derived from de novo gene birth, HGT and gene duplication, respectively, but 34 families only appear new due to selective retention in symbiotic species. The genes of gene families unique to ECM *Amanita* are upregulated during symbiosis and are likely functionally relevant to the symbiosis. The horizontally transferred gene encoding ACC deaminase is potentially crucial to the mutualistic relationship, possibly regulating the immune response in plant symbionts. Our findings suggest a new possibility for ECM evolution: the transition to ECM niche in fungi is not only driven by gene loss but by coincident new gene acquisition as well.

## Supplementary Material

Supplementary data are available at *Genome Biology and Evolution* online.

## Supplementary Material

evaa193_Supplementary_Data

## Notes

**Data deposition:** This project has been deposited at Dryad under the accession 10.5061/dryad.s4mw6m94f

## References

[evaa193-B1] Almagro Armenteros JJ, et al 2019 SignalP 5.0 improves signal peptide predictions using deep neural networks. Nat Biotechnol. 37(4):420–423. 30778233 10.1038/s41587-019-0036-z

[evaa193-B2] Almagro Armenteros JJ Sønderby CK Sønderby SK Nielsen H Winther O. 2017 DeepLoc: prediction of protein subcellular localization using deep learning. Bioinformatics 33(21):3387–3395. 29036616 10.1093/bioinformatics/btx431

[evaa193-B3] Altschul SF Gish W Miller W Myers EW Lipman DJ. 1990 Basic local alignment search tool. J Mol Biol. 215(3):403–410. 2231712 10.1016/S0022-2836(05)80360-2

[evaa193-B4] Andersson DI Jerlström-Hultqvist J Nasväll J. 2015 Evolution of new functions de novo and from preexisting genes. Cold Spring Harb Perspect Biol. 7(6):a017996. 26032716 10.1101/cshperspect.a017996 PMC4448608

[evaa193-B5] Arendsee Z, et al 2019 Fagin: synteny-based phylostratigraphy and finer classification of young genes. BMC Bioinformatics 20(1):440. 31455236 10.1186/s12859-019-3023-y PMC6712868

[evaa193-B6] Assis R Bachtrog D. 2013 Neofunctionalization of young duplicate genes in *Drosophila*. Proc Natl Acad Sci. 110(43):17409–17414. 24101476 10.1073/pnas.1313759110 PMC3808614

[evaa193-B7] Bansal MS Alm EJ Kellis M. 2012 Efficient algorithms for the reconciliation problem with gene duplication, horizontal transfer and loss. Bioinformatics 28(12):i283–291. 22689773 10.1093/bioinformatics/bts225 PMC3371857

[evaa193-B8] Bao D, et al 2013 Sequencing and comparative analysis of the straw mushroom (*Volvariella volvacea*) genome. PLoS One 8(3):e58294. 23526973 10.1371/journal.pone.0058294 PMC3602538

[evaa193-B9] Begun DJ Lindfors HA Kern AD Jones CD. 2007 Evidence for de novo evolution of testis-expressed genes in the *Drosophila yakuba*/*Drosophila erecta* clade. Genetics 176(2):1131–1137. 17435230 10.1534/genetics.106.069245 PMC1894579

[evaa193-B10] Bittleston LS Pierce NE Ellison AM Pringle A. 2016 Convergence in multispecies interactions. Trends Ecol Evol. 31(4):269–280. 26858111 10.1016/j.tree.2016.01.006

[evaa193-B11] Byrne KP Wolfe KH. 2007 Consistent patterns of rate asymmetry and gene loss indicate widespread neofunctionalization of yeast genes after whole-genome duplication. Genetics 175(3):1341–1350. 17194778 10.1534/genetics.106.066951 PMC1840088

[evaa193-B12] Cai J Zhao R Jiang H Wang W. 2008 De novo origination of a new protein-coding gene in *Saccharomyces cerevisiae*. Genetics 179(1):487–496. 18493065 10.1534/genetics.107.084491 PMC2390625

[evaa193-B13] Capella-Gutiérrez S Silla-Martínez JM Gabaldón T. 2009 TrimAl: a tool for automated alignment trimming in large-scale phylogenetic analyses. Bioinformatics 25:1972–1973. 19505945 10.1093/bioinformatics/btp348 PMC2712344

[evaa193-B14] Carvunis AR, et al 2012 Proto-genes and de novo gene birth. Nature 487(7407):370–374. 22722833 10.1038/nature11184 PMC3401362

[evaa193-B15] Casola C. 2018 From de novo to “*de nono*”: the majority of novel protein-coding genes identified with phylostratigraphy are old genes or recent duplicates. Genome Biol Evol. 10:2906–2918. 30346517 10.1093/gbe/evy231 PMC6239577

[evaa193-B16] Chaib de Mares M, et al 2015 Horizontal transfer of carbohydrate metabolism genes into ectomycorrhizal *Amanita*. New Phytol. 205(4):1552–1564. 25407899 10.1111/nph.13140

[evaa193-B17] Darriba D Taboada GL Doallo R Posada D. 2011 ProtTest 3: fast selection of best-fit models of protein evolution. Bioinformatics 27(8):1164–1165. 21335321 10.1093/bioinformatics/btr088 PMC5215816

[evaa193-B18] Donoghue MT Keshavaiah C Swamidatta SH Spillane C. 2011 Evolutionary origins of Brassicaceae specific genes in *Arabidopsis thaliana*. BMC Evol Biol. 11:47. 21332978 10.1186/1471-2148-11-47 PMC3049755

[evaa193-B19] Edgar RC. 2004 MUSCLE: multiple sequence alignment with high accuracy and high throughput. Nucleic Acids Res. 32(5):1792–1797. 15034147 10.1093/nar/gkh340 PMC390337

[evaa193-B20] Edgar RC. 2010 Search and clustering orders of magnitude faster than BLAST. Bioinformatics 26(19):2460–2461. 20709691 10.1093/bioinformatics/btq461

[evaa193-B21] Friesen TL, et al 2006 Emergence of a new disease as a result of interspecific virulence gene transfer. Nat Genet. 38(8):953–956. 16832356 10.1038/ng1839

[evaa193-B22] Gharib WH Robinson-Rechavi M. 2013 The branch-site test of positive selection is surprisingly robust but lacks power under synonymous substitution saturation and variation in GC. Mol Biol Evol. 30(7):1675–1686. 23558341 10.1093/molbev/mst062 PMC3684852

[evaa193-B23] Gluck-Thaler E Slot JC. 2015 Dimensions of horizontal gene transfer in eukaryotic microbial pathogens. PLoS Pathog. 11(10):e1005156. 26513155 10.1371/journal.ppat.1005156 PMC4626037

[evaa193-B24] Guindon S Perrière G. 2001 Intragenomic base content variation is a potential source of biases when searching for horizontally transferred genes. Mol Biol Evol. 18(9):1838–1840. 11504864 10.1093/oxfordjournals.molbev.a003972

[evaa193-B25] Hammesfahr B Odronitz F Mühlhausen S Waack S Kollmar M. 2013 GenePainter: a fast tool for aligning gene structures of eukaryotic protein families, visualizing the alignments and mapping gene structures onto protein structures. BMC Bioinformatics 14(1):77. 23496949 10.1186/1471-2105-14-77 PMC3605371

[evaa193-B26] Hatje K, et al 2011 Cross-species protein sequence and gene structure prediction with fine-tuned Webscipio 2.0 and Scipio. BMC Res Notes 4:265. 21798037 10.1186/1756-0500-4-265 PMC3162530

[evaa193-B27] Hess J, et al 2018 Rapid divergence of genome architectures following the origin of an ectomycorrhizal symbiosis in the genus *Amanita*. Mol Biol Evol. 35:2786–2804. 30239843 10.1093/molbev/msy179 PMC6231487

[evaa193-B28] Hess J Pringle A. 2014 The natural histories of species and their genomes: asymbiotic and ectomycorrhizal *Amanita* fungi. Adv Bot Res. 70:235–257.

[evaa193-B29] Honma M Smmomura T. 1978 Metabolism of 1-aminocyclopropane-1-carboxylic acid. Agric Biol Chem. 42(10):1825–1831.

[evaa193-B30] Huerta-Cepas J Dopazo J Gabaldón T. 2010 ETE: a python environment for tree exploration. BMC Bioinformatics 11(1):24. 20070885 10.1186/1471-2105-11-24 PMC2820433

[evaa193-B31] Husnik F McCutcheon JP. 2018 Functional horizontal gene transfer from bacteria to eukaryotes. Nat Rev Microbiol. 16(2):67–79. 29176581 10.1038/nrmicro.2017.137

[evaa193-B32] Jacox E Chauve C Szöllősi GJ Ponty Y Scornavacca C. 2016 EcceTERA: comprehensive gene tree-species tree reconciliation using parsimony. Bioinformatics 32(13):2056–2058. 27153713 10.1093/bioinformatics/btw105

[evaa193-B33] Karev GP Wolf YI Rzhetsky AY Berezovskaya FS Koonin EV. 2002 Birth and death of protein domains: a simple model of evolution explains power law behavior. BMC Evol Biol. 2(1):18. 12379152 10.1186/1471-2148-2-18 PMC137606

[evaa193-B34] Katoh K Misawa K Kuma K Miyata T. 2002 MAFFT: a novel method for rapid multiple sequence alignment based on fast Fourier transform. Nucleic Acids Res. 30(14):3059–3066. 12136088 10.1093/nar/gkf436 PMC135756

[evaa193-B35] Keeling PJ Palmer JD. 2008 Horizontal gene transfer in eukaryotic evolution. Nat Rev Genet. 9(8):605–618. 18591983 10.1038/nrg2386

[evaa193-B36] Kim D Langmead B Salzberg SL. 2015 HISAT: a fast spliced aligner with low memory requirements. Nat Methods 12(4):357–360. 25751142 10.1038/nmeth.3317 PMC4655817

[evaa193-B37] Kloppholz S Kuhn H Requena N. 2011 A secreted fungal effector of *Glomus intraradices* promotes symbiotic biotrophy. Curr Biol. 21(14):1204–1209. 21757354 10.1016/j.cub.2011.06.044

[evaa193-B38] Knowles DG McLysaght A. 2009 Recent de novo origin of human protein-coding genes. Genome Res. 19(10):1752–1759. 19726446 10.1101/gr.095026.109 PMC2765279

[evaa193-B39] Kohler A, et al 2015 Convergent losses of decay mechanisms and rapid turnover of symbiosis genes in mycorrhizal mutualists. Nat Genet. 47(4):410–415. 25706625 10.1038/ng.3223

[evaa193-B40] Krogh A Larsson B Von Heijne G Sonnhammer ELL. 2001 Predicting transmembrane protein topology with a hidden Markov model: application to complete genomes. J Mol Biol. 305(3):567–580. 11152613 10.1006/jmbi.2000.4315

[evaa193-B41] Li L Stoeckert CJ Roos DS. 2003 OrthoMCL: identification of ortholog groups for eukaryotic genomes. Genome Res. 13(9):2178–2189. 12952885 10.1101/gr.1224503 PMC403725

[evaa193-B42] Librado P Vieira FG Rozas J. 2012 BadiRate: estimating family turnover rates by likelihood-based methods. Bioinformatics 28(2):279–281. 22080468 10.1093/bioinformatics/btr623

[evaa193-B43] Long M VanKuren NW Chen S Vibranovski MD. 2013 New gene evolution: little did we know. Annu Rev Genet. 47(1):307–333. 24050177 10.1146/annurev-genet-111212-133301 PMC4281893

[evaa193-B44] Love MI Huber W Anders S. 2014 Moderated estimation of fold change and dispersion for RNA-seq data with DESeq2. Genome Biol. 15(12):550. 25516281 10.1186/s13059-014-0550-8 PMC4302049

[evaa193-B45] Loytynoja A Goldman N. 2005 From the cover: an algorithm for progressive multiple alignment of sequences with insertions. Proc Natl Acad Sci. 102(30):10557–10562. 16000407 10.1073/pnas.0409137102 PMC1180752

[evaa193-B47] Marcet-Houben M Gabaldón T. 2011 TreeKO: a duplication-aware algorithm for the comparison of phylogenetic trees. Nucleic Acids Res. 39(10):e66. 21335609 10.1093/nar/gkr087 PMC3105381

[evaa193-B48] Marri PR Golding GB. 2008 Gene amelioration demonstrated: the journey of nascent genes in bacteria. Genome 51(2):164–168. 18356951 10.1139/g07-105

[evaa193-B49] Matheny PB, et al 2006 Major clades of Agaricales: a multilocus phylogenetic overview. Mycologia. 98(6):982–995. 17486974 10.3852/mycologia.98.6.982

[evaa193-B50] Mayr E. 1963 Animal species and evolution. Cambridge (MA): Harvard University Press.

[evaa193-B51] Milde S, et al 2009 Characterization of taxonomically restricted genes in a phylum-restricted cell type. Genome Biol. 10(1):R8. 19161630 10.1186/gb-2009-10-1-r8 PMC2687796

[evaa193-B52] Moran NA Jarvik T. 2010 Lateral transfer of genes from fungi underlies carotenoid production in aphids. Science 328(5978):624–627. 20431015 10.1126/science.1187113

[evaa193-B53] Moyers BA Zhang J. 2015 Phylostratigraphic bias creates spurious patterns of genome evolution. Mol Biol Evol. 32(1):258–267. 25312911 10.1093/molbev/msu286 PMC4271527

[evaa193-B54] Murat C, et al 2018 Pezizomycetes genomes reveal the molecular basis of ectomycorrhizal truffle lifestyle. Nat Ecol Evol. 2(12):1956–1965. 30420746 10.1038/s41559-018-0710-4

[evaa193-B55] Nascimento FX Brígido C Glick BR Rossi MJ. 2016 The role of rhizobial ACC deaminase in the nodulation process of leguminous plants. Int J Agron. 2016:1–9.

[evaa193-B56] Ohno S. 1970 Evolution by gene duplication. New York: Springer-Verlag.

[evaa193-B57] Palmieri N Kosiol C Schlötterer C. 2014 The life cycle of *Drosophila* orphan genes. eLife 3:e01311. 24554240 10.7554/eLife.01311 PMC3927632

[evaa193-B58] Peter M, et al 2016 Ectomycorrhizal ecology is imprinted in the genome of the dominant symbiotic fungus *Cenococcum geophilum*. Nat Commun. 7:12662. 27601008 10.1038/ncomms12662 PMC5023957

[evaa193-B59] Pigliucci M. 2008 What, if anything, is an evolutionary novelty? Philos Sci. 75(5):887–898.

[evaa193-B60] Plett JM Daguerre Y, et al 2014 Effector MiSSP7 of the mutualistic fungus *Laccaria bicolor* stabilizes the *Populus* JAZ6 protein and represses jasmonic acid (JA) responsive genes. Proc Natl Acad Sci. 111(22):8299–8304. 24847068 10.1073/pnas.1322671111 PMC4050555

[evaa193-B61] Plett JM Khachane A, et al 2014 Ethylene and jasmonic acid act as negative modulators during mutualistic symbiosis between *Laccaria bicolor* and *Populus* roots. New Phytol. 202(1):270–286. 24383411 10.1111/nph.12655

[evaa193-B62] Pond SLK Frost SDW Muse SV. 2005 HyPhy: hypothesis testing using phylogenies. Bioinformatics 21(5):676–679. 15509596 10.1093/bioinformatics/bti079

[evaa193-B63] Potter SC, et al 2018 HMMER web server: 2018 update. Nucleic Acids Res. 46(W1):W200–204. 29905871 10.1093/nar/gky448 PMC6030962

[evaa193-B64] Price MN Dehal PS Arkin AP. 2010 FastTree 2—approximately maximum-likelihood trees for large alignments. PLoS One 5(3):e9490. 20224823 10.1371/journal.pone.0009490 PMC2835736

[evaa193-B65] Pulman JA Childs KL Sgambelluri RM Walton JD. 2016 Expansion and diversification of the MSDIN family of cyclic peptide genes in the poisonous agarics *Amanita phalloides* and *A. bisporigera*. BMC Genomics 17(1):1038. 27978833 10.1186/s12864-016-3378-7 PMC5159998

[evaa193-B66] Qiu H Cai G Luo J Bhattacharya D Zhang N. 2016 Extensive horizontal gene transfers between plant pathogenic fungi. BMC Biol. 14(1):41. 27215567 10.1186/s12915-016-0264-3 PMC4876562

[evaa193-B67] Rouanet C Nasser W. 2001 The PecM protein of the phytopathogenic bacterium *Erwinia chrysanthemi*, membrane topology and possible involvement in the efflux of the blue pigment indigoidine. J Mol Microbiol Biotechnol. 3(2):309–318. 11321588

[evaa193-B68] Ruiz-Orera J Verdaguer-Grau P Villanueva-Cañas JL Messeguer X Albà MM. 2018 Translation of neutrally evolving peptides provides a basis for de novo gene evolution. Nat Ecol Evol. 2(5):890–896. 29556078 10.1038/s41559-018-0506-6

[evaa193-B69] Saad R Cohanim AB Kosloff M Privman E. 2018 Neofunctionalization in ligand binding sites of ant olfactory receptors. Genome Biol Evol. 10(9):2490–2500. 29982411 10.1093/gbe/evy131 PMC6161762

[evaa193-B70] Schlötterer C. 2015 Genes from scratch—the evolutionary fate of de novo genes. Trends Genet. 31(4):215–219. 25773713 10.1016/j.tig.2015.02.007 PMC4383367

[evaa193-B71] Shimodaira H. 2002 An approximately unbiased test of phylogenetic tree selection. Syst Biol. 51(3):492–508. 12079646 10.1080/10635150290069913

[evaa193-B72] Shimodaira H Hasegawa M. 2001 CONSEL: for assessing the confidence of phylogenetic tree selection. Bioinformatics 17(12):1246–1247. 11751242 10.1093/bioinformatics/17.12.1246

[evaa193-B73] Slot JC. 2017 Fungal gene cluster diversity and evolution. Adv Genet. 100:141–178. 29153399 10.1016/bs.adgen.2017.09.005

[evaa193-B74] Smith MD, et al 2015 Less is more: an adaptive branch-site random effects model for efficient detection of episodic diversifying selection. Mol Biol Evol. 32(5):1342–1353. 25697341 10.1093/molbev/msv022 PMC4408413

[evaa193-B75] Smith S Read D. 2008 Mycorrhizal symbiosis, 3rd ed Cambridge (MA): Academic Press.

[evaa193-B76] Soanes D Richards TA. 2014 Horizontal gene transfer in eukaryotic plant pathogens. Annu Rev Phytopathol. 52(1):583–614. 25090479 10.1146/annurev-phyto-102313-050127

[evaa193-B77] Soshnikova N Dewaele R Janvier P Krumlauf R Duboule D. 2013 Duplications of Hox gene clusters and the emergence of vertebrates. Dev Biol. 378(2):194–199. 23501471 10.1016/j.ydbio.2013.03.004

[evaa193-B78] Spatafora JW, et al 2017 The fungal tree of life: from molecular systematics to genome-scale phylogenies. Microbiol Spectr. 5:5. 10.1128/microbiolspec.funk-0053-2016 PMC11687545 28917057

[evaa193-B79] Staehlin BM Gibbons JG Rokas A O’Halloran TV Slot JC. 2016 Evolution of a heavy metal homeostasis/resistance island reflects increasing copper stress in enterobacteria. Genome Biol Evol. 8:811–826. 26893455 10.1093/gbe/evw031 PMC4824010

[evaa193-B80] Stamatakis A. 2006 RAxML-VI-HPC: maximum likelihood-based phylogenetic analyses with thousands of taxa and mixed models. Bioinformatics 22(21):2688–2690. 16928733 10.1093/bioinformatics/btl446

[evaa193-B81] Stolzer M, et al 2012 Inferring duplications, losses, transfers and incomplete lineage sorting with nonbinary species trees. Bioinformatics 28(18):i409–415. 22962460 10.1093/bioinformatics/bts386 PMC3436813

[evaa193-B82] Tedersoo L May TW Smith ME. 2010 Ectomycorrhizal lifestyle in fungi: global diversity, distribution, and evolution of phylogenetic lineages. Mycorrhiza 20(4):217–263. 20191371 10.1007/s00572-009-0274-x

[evaa193-B83] Tofigh A Hallett M Lagergren J. 2011 Simultaneous identification of duplications and lateral gene transfers. IEEE/ACM Trans Comput Biol Bioinform. 8(2):517–535. 21233529 10.1109/TCBB.2010.14

[evaa193-B84] Vakirlis N, et al 2018 A molecular portrait of de novo genes in yeasts. Mol Biol Evol. 35(3):631–645. 29220506 10.1093/molbev/msx315 PMC5850487

[evaa193-B85] van der Nest MA, et al 2014 Draft genomes of *Amanita jacksonii*, *Ceratocystis albifundus*, *Fusarium circinatum*, *Huntiella omanensis*, *Leptographium procerum*, *Rutstroemia sydowiana*, and *Sclerotinia echinophila*. IMA Fungus 5(2):472–486. 10.5598/imafungus.2014.05.02.11 PMC4329328 25734036

[evaa193-B86] van Dongen S. 2000. Graph clustering by flow simulation. doi:10.1016/j.cosrev.2007.05.001.

[evaa193-B87] Varga T, et al 2019 Megaphylogeny resolves global patterns of mushroom evolution. Nat Ecol Evol. 3(4):668–678. 30886374 10.1038/s41559-019-0834-1 PMC6443077

[evaa193-B88] Västermark Å Almén MS Simmen MW Fredriksson R Schiöth HB. 2011 Functional specialization in nucleotide sugar transporters occurred through differentiation of the gene cluster EamA (DUF6) before the radiation of Viridiplantae. BMC Evol Biol. 11:123. 21569384 10.1186/1471-2148-11-123 PMC3111387

[evaa193-B89] Villanueva-Cañas JL, et al 2017 New genes and functional innovation in mammals. Genome Biol Evol. 9:1886–1900. 28854603 10.1093/gbe/evx136 PMC5554394

[evaa193-B90] Wisecaver JH Slot JC Rokas A. 2014 The evolution of fungal metabolic pathways. PLoS Genet. 10(12):e1004816. 25474404 10.1371/journal.pgen.1004816 PMC4256263

[evaa193-B91] Wolfe BE Tulloss RE Pringle A. 2012 The irreversible loss of a decomposition pathway marks the single origin of an ectomycorrhizal symbiosis. PLoS One 7(7):e39597. 22815710 10.1371/journal.pone.0039597 PMC3399872

[evaa193-B92] Wu B Knudson A. 2018 Tracing the de novo origin of protein-coding genes in yeast. mBio. 9(4):e01024–18. 30065088 10.1128/mBio.01024-18 PMC6069113

[evaa193-B93] Yang Z. 2007 PAML 4: phylogenetic analysis by maximum likelihood. Mol Biol Evol. 24(8):1586–1591. 17483113 10.1093/molbev/msm088

[evaa193-B94] Zhang J. 2003 Evolution by gene duplication: an update. Trends Ecol Evol. 18(6):292–298.

