# Supplementary material for: De Novo Gene Birth, Horizontal Gene Transfer, and Gene Duplication as Sources of New Gene Families Associated with the Origin of Symbiosis in Amanita
Source: Genome Biol Evol. 2020 Sep 14;12(11):2168–82. doi: 10.1093/gbe/evaa193 (PMC7674699; doi:10.1093/gbe/evaa193)
Supplement: evaa193_Supplementary_Data [file evaa193_supplementary_data.zip › S3,4,6,7,8.docx]

**Supplementary file 3**

**Family 2813**

**Monophyletic Ascomycota**

((Amamu0_2813.1,Amabr0_12239.1,Amapo0_5779.5,Phlbr1_152555,Bjead1_168289,Phlgi1_103031,Phaca1_195663,Phchr2_3014243,Rhomi1_191880,Daequ1_281192,Laesu1_758888,Cersu1_116177,Obbri1_797302,Dicsq1_23626,Gansp1_111552,Wolco1_91398,Pycci1_3629,Polar1_594463,Polbr1_1488099,Dacsp1_115900,Calvi1_506161,Calco1_538057,Calco1_426110,Calco1_551524,Glotr1_47697,Helsul1_453149,Neole1_1133107,Jaaar1_57340,Sphst1_172091,Sphst1_188037,Cvar1_XP_005847727.1,Mneg1_XP_013905929.1,Sfum1_YP_845036.1,SNBC1_YP_001358307.1,Gura1_YP_001229178.1,Gbem1_YP_002138394.1,GM211_YP_003022429.1,Tulca1_180015,Tulca1_22150,Exoaq1_A1O902500T0,Exoxe01_4763,Eder_05008T0,Capco1_A1O108725T0,Capep1_A1O308111T0,Exool02_1146,Claps1_A1O500117T0,Claca1_G64704867T0,Claye1_A1O703512T0),(Aurde31_1410154,Xylhe1_241921,Aurpuvarpul1_55426,Aurpuvarmel1_73296,Aurpuvarnam1_60816,Aurpuvarsub1_1724,Rhyru1_2783,Cenge3_704213,Corca1_448707,Conap1_W9701086T0,Ascim1_411344,Dipse1_00002088,Botdo1_13272,Sodal1_77672,Oidma1_103493,Lipst1_192626,Pdes_1029,Pyrco1_12059,Eutla1_807,DalEC121_72284,HypCO2751_20134,HypEC381_1889,Podan2_10113,Phaal1_5762,Ascsa1_9468,Conlig1_568514,Metac1_264,Metan1_8199,Trias1_57252,Triat2_295820,Triha1_110430,TriviGv2982_224223,Trilo3_1437611,Trire2_60370,TrireRUTC301_76961,Necha2_50395,Fusoxii5_FOIG06758T0,Fusoxhdv247_FOVG08419T0,Fusoxphw815_FOQG06396T0,Fusoxcotton_FOTG09913T0,Fusoxphw808_FOPG03254T0,Fusox1_12894,Fusoxfspmelonis_FOMG03045T0,Fusoxcl57_FOCG11236T0,Fusoxfo47_FOZG02968T0,Fusoxmn25_FOWG13858T0,Fusoxnrrl32931_FOYG00658T0,Aspoch1_531825,Aspnid1_1995,Aspsy1_89091,Aspve1_54294,Aspac1_40153,Aspca3_166220,Aspbr1_76234,AspniDSM1_168803,AspniNRRL31_10451,Asptu1_112558,Aspfo1_139765,Aspka11_12955,Terbo1_8443,Aspte1_6609,Aspcam1_236865,Aspste1_466210,Talma12_4944,Aspfl1_37088,Aspor1_300,Aspwe1_26045,Aspgl1_33272,Eurhe1_378221,Aspzo1_131161,Aspnov1_426216,Aspcl1_7861,Aspfu1_556,AspfuA11631_99773,Neofi1_3249,Aspnov1_421219,Aspnov1_436321,Pendi1_5595,Pench1_76846,PenchWisc11_138979,Penox1_2718,Mcan_00935T0,Micca1_952,Triru1_493,Artbe1_4764,Triver1_7339,Cocim1_9526,Uncre1_1917,Parbr1_4021,Bder_05024T0,Hisca1_212));

**Family 7854**

**Monophyletic Agaricales**

((Amabr0_1754.1,Amamu0_7854.1,Amapo0_1540.1,Armme11_13724,Armme11_6222,Armos1_AROS17743g,Armost1_963400,Lacbi2_489120),(Dacsp1_24395,Clagr3_2553,Gaegr1_GGTG00347T0,Cadsp1_652755,Marbr1_2435,Talma12_9993,Cocmi1_39783,Cenge3_707698,Leppa1_384580,Aspwe1_25307,Pench1_35713,Pendi1_5983,Calco1_216433,Calvi1_595939,Calvi1_595940,Calvi1_595942,Aspnid1_1953,Aspsy1_90883,Aspve1_153772,Conlig1_635465,Phisc1_688850,Aspnov1_373009,Aspsy1_133430,Oidma1_21636,Neofi1_311,Aspfu1_5870,AspfuA11631_106138,Leppa1_298334,Metac1_7638,Eder_02102T0,Lipst1_5090,Aurpuvarpul1_319566,Melbi2_536963,Melva1_505345,Penox1_3618,Aspcl1_4865,Neofi1_804,Aspgl1_46247,Aspfu1_4680,AspfuA11631_108930,Aspste1_505422,Aspnid1_7274,Aspoch1_497273,Aspnov1_506813,Bder_04476T0,Pendi1_5050,Aspca3_132587,Asptu1_67160,Aspwe1_43468));

**Monophyletic Ascomycota**

((Calco1_216433,Calvi1_595939,Calvi1_595940,Calvi1_595942,Amamu0_7854.1,Amabr0_1754.1,Amapo0_1540.1),(Armme11_13724,Armme11_6222,Armos1_AROS17743g,Armost1_963400,Aspwe1_25307,Pench1_35713,Pendi1_5983,Talma12_9993,Aspnid1_1953,Aspsy1_90883,Aspve1_153772,Conlig1_635465,Phisc1_688850,Aspnov1_373009,Aspsy1_133430,Oidma1_21636,Neofi1_311,Aspfu1_5870,AspfuA11631_106138,Dacsp1_24395,Clagr3_2553,Gaegr1_GGTG00347T0,Cadsp1_652755,Marbr1_2435,Lacbi2_489120,Cocmi1_39783,Cenge3_707698,Leppa1_384580,Leppa1_298334,Metac1_7638,Eder_02102T0,Lipst1_5090,Aurpuvarpul1_319566,Melbi2_536963,Melva1_505345,Penox1_3618,Aspcl1_4865,Neofi1_804,Aspgl1_46247,Aspfu1_4680,AspfuA11631_108930,Aspste1_505422,Aspnid1_7274,Aspoch1_497273,Aspnov1_506813,Bder_04476T0,Pendi1_5050,Aspca3_132587,Asptu1_67160,Aspwe1_43468));

**Family 10418**

**Monophyletic Agaricales**

((Amabr0_9971.1,Amamu0_10418.1,Amapo0_1269.1,Armme11_7775,Armos1_AROS17769g,Armost1_963440,Gymlu1_43251,Monpe11_81883),(Cocim1_698,Triru1_3236,Artbe1_6636,Triver1_2575,Mcan_07709T0,Micca1_7910,Artol1_3805,Monha1_1313,Aspwe1_113062,Aspgl1_137907,Eurhe1_465119,Pendi1_2932,Pench1_73093,PenchWisc11_149700,Aspnov1_436391,Neofi1_1254,Aspfu1_1733,AspfuA11631_100925,Aspac1_54302,Aspste1_75287,Aspfl1_31331,Aspor1_7680,Aspca3_509234,Aspbr1_58452,Aspfo1_51703,Aspka11_21458,Asptu1_56472,Aspni7_1158674,AspniNRRL31_6331,Cylto1_385789,Denbi1_820086,Denbi1_863491,Glalo1_1525,Phisc1_645306,Ascsa1_8399,Cadsp1_652643,Marbr1_5607,Phieu1_HMPREF154110150T0,Exoaq1_A1O901011T0,Exome1_759216955,Exool02_6670,Claps1_A1O512257T0,Claca1_G64701688T0,Claye1_A1O701606T0,Spoth2_2300602,Thite2_2106728,Neucr2_7043,Neucrtrp31_7043,Neutemata1_155197,NeutematA2_77478,Phaal1_8068,Beaba1_4627,Cormi1_5149,Metac1_8292,Metan1_311,Trilo3_1448102,Trire2_5107,TrireRUTC301_86627,Trias1_62485,Triat2_238874,Triha1_8260,TriviGv2982_66237,Ilysp1_1582239,Necha2_33030,Fusve1_12439,Fusfu1_2350,Fusgr1_5854,Fusoxnrrl32931_FOYG05493T0,Fusoxcl57_FOCG03277T0,Fusoxphw815_FOQG10272T0,Fusoxfspmelonis_FOMG12609T0,Fusoxhdv247_FOVG05263T0,Fusoxmn25_FOWG01475T0,Fusoxii5_FOIG07992T0,Fusoxcotton_FOTG04980T0,Fusoxfo47_FOZG06569T0,Fusox1_1874,Fusoxphw808_FOPG05251T0,DalEC121_187808,HypCI4A1_10470,HypCO2751_399581,HypEC381_393507,Maggr1_117176,Gaegr1_GGTG11668T0,Magpo1_022260,Verda1_7445,Sodal1_311255,Colhi1_11211,Colgr1_283,Colhi1_5473,Eutla1_7267,Ophpc1_601,Phaal1_6230,Cenge3_638826,Exoaq1_A1O901767T0,Capep1_A1O306411T0,Capco1_A1O105108T0,Eder_02133T0,Aspzo1_155227,Aspnid1_6993,Aspsy1_162613,Aspve1_34576,Oidma1_171561,Aspoch1_512133,Oidma1_43316,Talma12_8753));

**Monophyletic Ascomycota**

((Amamu0_10418.1,Amabr0_9971.1,Amapo0_1269.1,Denbi1_820086,Denbi1_863491,Cylto1_385789,Gymlu1_43251,Monpe11_81883,Armme11_7775,Armos1_AROS17769g,Armost1_963440),(Artol1_3805,Monha1_1313,Aspwe1_113062,Aspgl1_137907,Eurhe1_465119,Pendi1_2932,Pench1_73093,PenchWisc11_149700,Aspnov1_436391,Neofi1_1254,Aspfu1_1733,AspfuA11631_100925,Aspac1_54302,Aspste1_75287,Aspfl1_31331,Aspor1_7680,Aspca3_509234,Aspbr1_58452,Aspfo1_51703,Aspka11_21458,Asptu1_56472,Aspni7_1158674,AspniNRRL31_6331,Cocim1_698,Triru1_3236,Artbe1_6636,Triver1_2575,Mcan_07709T0,Micca1_7910,Glalo1_1525,Phisc1_645306,Ascsa1_8399,Cadsp1_652643,Marbr1_5607,Phieu1_HMPREF154110150T0,Exoaq1_A1O901011T0,Exome1_759216955,Exool02_6670,Claps1_A1O512257T0,Claca1_G64701688T0,Claye1_A1O701606T0,Spoth2_2300602,Thite2_2106728,Neucr2_7043,Neucrtrp31_7043,Neutemata1_155197,NeutematA2_77478,Phaal1_8068,Beaba1_4627,Cormi1_5149,Metac1_8292,Metan1_311,Trilo3_1448102,Trire2_5107,TrireRUTC301_86627,Trias1_62485,Triat2_238874,Triha1_8260,TriviGv2982_66237,Ilysp1_1582239,Necha2_33030,Fusve1_12439,Fusfu1_2350,Fusgr1_5854,Fusoxnrrl32931_FOYG05493T0,Fusoxcl57_FOCG03277T0,Fusoxphw815_FOQG10272T0,Fusoxfspmelonis_FOMG12609T0,Fusoxhdv247_FOVG05263T0,Fusoxmn25_FOWG01475T0,Fusoxii5_FOIG07992T0,Fusoxcotton_FOTG04980T0,Fusoxfo47_FOZG06569T0,Fusox1_1874,Fusoxphw808_FOPG05251T0,DalEC121_187808,HypCI4A1_10470,HypCO2751_399581,HypEC381_393507,Maggr1_117176,Gaegr1_GGTG11668T0,Magpo1_022260,Verda1_7445,Sodal1_311255,Colhi1_11211,Colgr1_283,Colhi1_5473,Eutla1_7267,Ophpc1_601,Phaal1_6230,Cenge3_638826,Exoaq1_A1O901767T0,Capep1_A1O306411T0,Capco1_A1O105108T0,Eder_02133T0,Aspzo1_155227,Aspnid1_6993,Aspsy1_162613,Aspve1_34576,Oidma1_171561,Aspoch1_512133,Oidma1_43316,Talma12_8753));

**Family 11987**

**Monophyletic Agaricales**

((Galma1_80567,Lacam2_506929,Lacbi2_303990,Lacam2_506958,Lacbi2_330257,Amamu0_7928.1,Amabr0_12118.1,Amapo0_7770.2,Amamu0_11987.1,Amamu0_12010.1,Amamu0_11990.1,Amamu0_11994.1),(Aurpuvarmel1_62388,Cenge3_688138,Bauco1_27030,Psean11_80352,Tilan2_294968,Leppa1_401858,Aspoch1_343449,Oidma1_187792,Lipst1_91476,Thite2_2071939,Phisc1_688223,Melbi2_585646,Rhier1_607387,Capep1_A1O300944T0,Oidma1_43381,Aspoch1_534248,Aspor1_6737,Aspwe1_186923,Aspnov1_466562,Aspfu1_2023,AspfuA11631_101211,Talma12_2420,Amore1_146058,Pdes_2130,Amore1_98752,Lipst1_1422,Claps1_A1O503213T0,Exoxe01_2421,Aspbr1_47589,Grocl1_1640,Conlig1_279382,Neucrtrp31_1272546,Neucr2_5191,Neucr2_5192,Neutemata1_143898,NeutematA2_116203,Cormi1_187,Metac1_7785,Metan1_1204,Trilo3_1440262,Trire2_68973,TrireRUTC301_89357,Triha1_507960,TriviGv2982_55087,Trias1_58549,Triat2_302876,Jaaar1_142981,Jaaar1_68699,Aciri1iso_57042,Dotse1_161013,Melva1_485764,Phisc1_600076,Oidma1_120809,Phisc1_749160,Lipst1_333289,Lipst1_2005,Lipst1_5471,Lipst1_6103,Lipst1_94481,Triol1_285389,Rhoba1_29715,Rhosp1_33869,Dacsp1_87382,Paxin1_176637,Hydpi2_99288,Pilcr1_822271,Sclci1_145416,Pismi1_568931,Pisti1_160331,Debha1_29,Yarli1_65985,Yarli1_69246,Walse1_66883,Walic1_2852,Walic1_4498,Fusve1_8367,Fusoxii5_FOIG09622T0,Fusoxnrrl32931_FOYG15073T0,Fusoxcotton_FOTG11264T0,Fusoxhdv247_FOVG13920T0,Fusoxphw808_FOPG14791T0,Fusoxphw815_FOQG17568T0,Fusoxhdv247_FOVG07023T1,Fusoxfspmelonis_FOMG07548T1,Fusoxnrrl32931_FOYG08952T1,Fusoxfo47_FOZG10258T1,Fusoxmn25_FOWG14778T1,Fusoxcl57_FOCG05110T1,Fusoxcotton_FOTG11714T1,Fusoxphw808_FOPG13552T1,Fusoxii5_FOIG09093T1,Fusoxphw815_FOQG17263T1,Trias1_200828,Triat2_44629,Aciri1iso_329780,Aurpuvarmel1_65576,Aurpuvarnam1_52888,Aurpuvarpul1_298259,Grocl1_1786,Oidma1_134325,Metac1_1,Dacsp1_114495,Calco1_519528,Calvi1_566837,Mrub1_YP_003506479.1,PJDR1_YP_003011931.1,Ccle1_XP_006438893.1,Mtru1_XP_003607787.2,Obra1_XP_015698649.1,Zmay1_NP_001136973.1,Acom1_XP_020101512.1,Psme1_PSME_00004540-RA,Zjuj1_XP_015889874.1,Natt1_XP_019234848.1,Nsyl1_XP_009794994.1,Ntab1_XP_016435524.1,Fves1_XP_011459641.1,Esal1_XP_006409435.1,Pmum1_XP_008237013.1,Thas1_XP_010530813.1,Mtru1_XP_013442399.1,Lang1_XP_019431773.1,Lang1_XP_019454391.1,Ccaj1_XP_020202624.1,Gmax1_NP_001242198.1,Gmax1_XP_006594791.1,Pvul1_XP_007149618.1,Vang1_XP_017425093.1,Pvul1_XP_007155070.1,Pdac1_XP_008795838.1,Bvul2_XP_010696577.1,Bvul2_XP_010696569.1,Bvul2_XP_010696573.1,Bvul2_XP_019108251.1,Bvul2_XP_019108255.1,Jreg1_XP_018826789.1,Egra1_XP_018725263.1,Egra1_XP_010047263.1,Egra1_XP_010047265.1,Egra1_XP_010047261.1,Egra1_XP_010047262.1,Egra1_XP_010047264.1,Tcac1_XP_007011812.2,Tcac1_XP_017983506.1,Grai1_XP_012451812.1,Grai1_XP_012451813.1,Garb1_XP_017643213.1,Garb1_XP_017643215.1,Ghir1_XP_016754945.1,Ghir1_XP_016754946.1,Cvar1_XP_005848257.1,Vang1_XP_017407755.1,Vrad1_XP_014521676.1,Vrad1_XP_014521677.1,Ccaj1_XP_020228642.1,Cari1_XP_004502218.1,Cari1_XP_012571804.1,Pper1_XP_007222544.2,Pper1_XP_020418213.1,Pdac1_XP_008794938.2,Dcat1_XP_020687804.1,Dcat1_XP_020687814.1,Pequ1_XP_020575023.1,Pequ1_XP_020575024.1,Egra1_XP_010026022.1,Jreg1_XP_018840696.1,Jreg1_XP_018840697.1,Jreg1_XP_018840698.1,Ccaj1_XP_020227600.1,Lang1_XP_019450089.1,Lang1_XP_019450090.1,Pvul1_XP_007140628.1,Vang1_XP_017416155.1,Vrad1_XP_014496525.1,Ppat1_XP_001761645.1,Aoff1_XP_020256839.1,Aoff1_XP_020273354.1,Egut1_XP_012827493.1,Alyr1_XP_002883248.1,Vvin1_XP_002278488.2,Tcac1_XP_007020894.2,Zjuj1_XP_015898525.1,Lang1_XP_019443086.1,Pvul1_XP_007146445.1,Vang1_XP_017434932.1,Vrad1_XP_014517584.1,Lang1_XP_019435499.1,Lang1_XP_019435506.1,Mtru1_XP_003617509.1,Cari1_XP_004517048.1,Cari1_XP_004517049.1,Gmax1_NP_001239628.1,Gmax1_NP_001239671.1,Gmax1_XP_014621970.1,Pvul1_XP_007142266.1,Vang1_XP_017430795.1,Vrad1_XP_014504984.1,Atau1_XP_020174643.1,Atri1_XP_020527269.1,Atri1_XP_006851491.1,Atri1_XP_020527268.1,Mdom1_XP_008387079.2,Pxb1_XP_009374738.1,Pmum1_XP_008241255.1,Pper1_XP_007204390.1,Pabi1_MA_491198g0010,Psme1_PSME_00023345-RA,Acom1_XP_020106641.1,Peup1_XP_011044864.1,Csat1_XP_010515756.1,Csat1_XP_010426906.1,Csat1_XP_010426907.1,Alyr1_XP_002877889.1,Atha1_NP_190850.2,Crub1_XP_006291164.1,Esal1_XP_006403765.1,Sind1_XP_011094368.1,Jreg1_XP_018807738.1,Jreg1_XP_018813642.1,Fves1_XP_004302999.1,Pmum1_XP_008241257.1,Mnot1_XP_010101590.1,Mtru1_XP_013468010.1,Lang1_XP_019454500.1,Ccaj1_XP_020212561.1,Gmax1_XP_003521454.2,Pvul1_XP_007162887.1,Vang1_XP_017419013.1,Vrad1_XP_014496019.1));

**Monophyletic Ascomycota**

((Walse1_66883,Walic1_2852,Walic1_4498,Yarli1_65985,Yarli1_69246,Debha1_29,Lipst1_333289,Lipst1_2005,Lipst1_5471,Lipst1_6103,Lipst1_94481,Triol1_285389,Rhoba1_29715,Rhosp1_33869,Dacsp1_87382,Paxin1_176637,Hydpi2_99288,Pilcr1_822271,Sclci1_145416,Pismi1_568931,Pisti1_160331,Aciri1iso_57042,Dotse1_161013,Melva1_485764,Phisc1_600076,Oidma1_120809,Phisc1_749160,Amore1_146058,Pdes_2130,Amore1_98752,Lipst1_1422,Claps1_A1O503213T0,Exoxe01_2421,Aspbr1_47589,Grocl1_1640,Conlig1_279382,Neucrtrp31_1272546,Neucr2_5191,Neucr2_5192,Neutemata1_143898,NeutematA2_116203,Cormi1_187,Metac1_7785,Metan1_1204,Trilo3_1440262,Trire2_68973,TrireRUTC301_89357,Triha1_507960,TriviGv2982_55087,Trias1_58549,Triat2_302876,Aurpuvarmel1_62388,Jaaar1_142981,Jaaar1_68699,Galma1_80567,Lacam2_506929,Lacbi2_303990,Lacam2_506958,Lacbi2_330257,Cenge3_688138,Bauco1_27030,Psean11_80352,Tilan2_294968,Leppa1_401858,Aspoch1_343449,Oidma1_187792,Lipst1_91476,Thite2_2071939,Phisc1_688223,Melbi2_585646,Rhier1_607387,Capep1_A1O300944T0,Oidma1_43381,Aspoch1_534248,Aspor1_6737,Aspwe1_186923,Aspnov1_466562,Aspfu1_2023,AspfuA11631_101211,Talma12_2420,Fusve1_8367,Fusoxii5_FOIG09622T0,Fusoxnrrl32931_FOYG15073T0,Fusoxcotton_FOTG11264T0,Fusoxhdv247_FOVG13920T0,Fusoxphw808_FOPG14791T0,Fusoxphw815_FOQG17568T0,Fusoxhdv247_FOVG07023T1,Fusoxfspmelonis_FOMG07548T1,Fusoxnrrl32931_FOYG08952T1,Fusoxfo47_FOZG10258T1,Fusoxmn25_FOWG14778T1,Fusoxcl57_FOCG05110T1,Fusoxcotton_FOTG11714T1,Fusoxphw808_FOPG13552T1,Fusoxii5_FOIG09093T1,Fusoxphw815_FOQG17263T1,Trias1_200828,Triat2_44629,Aciri1iso_329780,Aurpuvarmel1_65576,Aurpuvarnam1_52888,Aurpuvarpul1_298259,Grocl1_1786,Oidma1_134325,Metac1_1,Dacsp1_114495,Calco1_519528,Calvi1_566837),(Amamu0_7928.1,Amabr0_12118.1,Amapo0_7770.2,Amamu0_11987.1,Amamu0_12010.1,Amamu0_11990.1,Amamu0_11994.1,Mrub1_YP_003506479.1,PJDR1_YP_003011931.1,Ccle1_XP_006438893.1,Mtru1_XP_003607787.2,Obra1_XP_015698649.1,Zmay1_NP_001136973.1,Acom1_XP_020101512.1,Psme1_PSME_00004540-RA,Zjuj1_XP_015889874.1,Natt1_XP_019234848.1,Nsyl1_XP_009794994.1,Ntab1_XP_016435524.1,Fves1_XP_011459641.1,Esal1_XP_006409435.1,Pmum1_XP_008237013.1,Thas1_XP_010530813.1,Mtru1_XP_013442399.1,Lang1_XP_019431773.1,Lang1_XP_019454391.1,Ccaj1_XP_020202624.1,Gmax1_NP_001242198.1,Gmax1_XP_006594791.1,Pvul1_XP_007149618.1,Vang1_XP_017425093.1,Pvul1_XP_007155070.1,Pdac1_XP_008795838.1,Bvul2_XP_010696577.1,Bvul2_XP_010696569.1,Bvul2_XP_010696573.1,Bvul2_XP_019108251.1,Bvul2_XP_019108255.1,Jreg1_XP_018826789.1,Egra1_XP_018725263.1,Egra1_XP_010047263.1,Egra1_XP_010047265.1,Egra1_XP_010047261.1,Egra1_XP_010047262.1,Egra1_XP_010047264.1,Tcac1_XP_007011812.2,Tcac1_XP_017983506.1,Grai1_XP_012451812.1,Grai1_XP_012451813.1,Garb1_XP_017643213.1,Garb1_XP_017643215.1,Ghir1_XP_016754945.1,Ghir1_XP_016754946.1,Cvar1_XP_005848257.1,Vang1_XP_017407755.1,Vrad1_XP_014521676.1,Vrad1_XP_014521677.1,Ccaj1_XP_020228642.1,Cari1_XP_004502218.1,Cari1_XP_012571804.1,Pper1_XP_007222544.2,Pper1_XP_020418213.1,Pdac1_XP_008794938.2,Dcat1_XP_020687804.1,Dcat1_XP_020687814.1,Pequ1_XP_020575023.1,Pequ1_XP_020575024.1,Egra1_XP_010026022.1,Jreg1_XP_018840696.1,Jreg1_XP_018840697.1,Jreg1_XP_018840698.1,Ccaj1_XP_020227600.1,Lang1_XP_019450089.1,Lang1_XP_019450090.1,Pvul1_XP_007140628.1,Vang1_XP_017416155.1,Vrad1_XP_014496525.1,Ppat1_XP_001761645.1,Aoff1_XP_020256839.1,Aoff1_XP_020273354.1,Egut1_XP_012827493.1,Alyr1_XP_002883248.1,Vvin1_XP_002278488.2,Tcac1_XP_007020894.2,Zjuj1_XP_015898525.1,Lang1_XP_019443086.1,Pvul1_XP_007146445.1,Vang1_XP_017434932.1,Vrad1_XP_014517584.1,Lang1_XP_019435499.1,Lang1_XP_019435506.1,Mtru1_XP_003617509.1,Cari1_XP_004517048.1,Cari1_XP_004517049.1,Gmax1_NP_001239628.1,Gmax1_NP_001239671.1,Gmax1_XP_014621970.1,Pvul1_XP_007142266.1,Vang1_XP_017430795.1,Vrad1_XP_014504984.1,Atau1_XP_020174643.1,Atri1_XP_020527269.1,Atri1_XP_006851491.1,Atri1_XP_020527268.1,Mdom1_XP_008387079.2,Pxb1_XP_009374738.1,Pmum1_XP_008241255.1,Pper1_XP_007204390.1,Pabi1_MA_491198g0010,Psme1_PSME_00023345-RA,Acom1_XP_020106641.1,Peup1_XP_011044864.1,Csat1_XP_010515756.1,Csat1_XP_010426906.1,Csat1_XP_010426907.1,Alyr1_XP_002877889.1,Atha1_NP_190850.2,Crub1_XP_006291164.1,Esal1_XP_006403765.1,Sind1_XP_011094368.1,Jreg1_XP_018807738.1,Jreg1_XP_018813642.1,Fves1_XP_004302999.1,Pmum1_XP_008241257.1,Mnot1_XP_010101590.1,Mtru1_XP_013468010.1,Lang1_XP_019454500.1,Ccaj1_XP_020212561.1,Gmax1_XP_003521454.2,Pvul1_XP_007162887.1,Vang1_XP_017419013.1,Vrad1_XP_014496019.1));

**Family 12806**

**Monophyletic Agaricales**

((Armme11_2624,Galma1_59305,Schco3_2489244,Armme11_2344,Amapo0_3172.1,Amabr0_14327.1,Amabr0_7709.1,Amamu0_12797.1,Amamu0_12806.1,Amamu0_7561.1),(Tilan2_249267,Punst1_124096,Helsul1_1629573,Neole1_1054711,Dacsp1_108877,Calco1_463966,Calvi1_126822,Sebve1_61343,Tulca1_220580,Sphst1_168580,Phaca1_256164,Phchr2_3001131,Fomme1_145373,Dicsq1_177432,Rhosp1_3647,Pucgr1_23743,Klula1_3451,Ascru1_74553,Calb_04312,Picst3_44393,Ppat1_XP_001755806.1,Pabi1_MA_362355g0010,Psme1_PSME_00030339-RA,Egui1_XP_010925634.1,Pdac1_XP_008789985.1,Cari1_XP_004514648.1,Cari1_XP_004514650.1,Natt1_XP_019249491.1,Nsyl1_XP_009798852.1,Ntab1_XP_016456661.1,Bnap1_XP_013736281.1,Brap1_XP_009135412.1,Garb1_XP_017617963.1,Ghir1_XP_016676131.1,Pila1_PILAlq_041310-RA,Pita1_PITA_000037874-RA,Atau1_XP_020151510.1,Sbic1_XP_002441054.1,Egui1_XP_010921004.1,Dcat1_XP_020685699.1,Pequ1_XP_020585972.1,Bvul2_XP_010693058.1,Inil1_XP_019151362.1,Cann1_XP_016547742.1,Cann1_XP_016564113.1,Natt1_XP_019230611.1,Ntom1_XP_009631854.1,Ntab1_XP_016471998.1,Ntom1_XP_009605678.1,Natt1_XP_019258060.1,Nsyl1_XP_009765120.1,Ntab1_XP_016475074.1,Jcur1_XP_012086876.1,Garb1_XP_017643464.1,Ghir1_XP_016681672.1,Mtru1_XP_003616684.1,Ccaj1_XP_020225890.1,Vang1_XP_017430394.1,Vrad1_XP_014504725.1,Jreg1_XP_018821797.1,Alyr1_XP_002873210.1,Atha1_NP_568155.1,Crub1_XP_006290079.1,Csat1_XP_010452449.1,Csat1_XP_010491079.1,Esal1_XP_006399009.1,Rsat1_XP_018476869.1,Rsat1_XP_018476870.1,Bole1_XP_013628953.1,Bnap1_XP_013724111.1,Brap1_XP_009130883.1,Pyrco1_5592,Morco1_524827,Morimp1_550840,Lipst1_50435,Artol1_4376,Monha1_1220,Glalo1_12150,Cadsp1_492036,Phisc1_581373,Melva1_529130,Melbi2_572993,Rhier1_600383,HypCI4A1_321098,HypCO2751_17521,HypEC381_395331,Melbi2_696696,Phisc1_706092,Cadsp1_350273,Glalo1_5527,Clagr3_4470,Aspzo1_105929,Aspzo1_26514,Aurpuvarpul1_291524,Aurpuvarsub1_102588,Aurpuvarmel1_13202,Aurpuvarnam1_86426,Parbr1_3036,Bder_01754T0,Hisca1_5375,Talma12_829,Exigl1_615646,Trave1_110824,Aspgl1_119608,Eurhe1_414300,Penox1_2509,Pendi1_5483,Pench1_76464,PenchWisc11_140476,Aspwe1_26209,Aspcl1_3624,Aspfu1_7733,AspfuA11631_106205,Aspnov1_404651,Neofi1_9562,Aspnid1_10092,Aspve1_133004,Aspor1_2736,Aspoch1_461782,Aspac1_28147,Aspca3_404216,Aspca3_45444,Asptu1_49841,Aspfo1_63878,Aspka11_21515,Aspbr1_57866,AspniDSM1_158324,Aspni7_1186485,AspniNRRL31_8553));

**Monophyletic Ascomycota**

((Lipst1_50435,Artol1_4376,Monha1_1220,Glalo1_12150,Cadsp1_492036,Phisc1_581373,Melva1_529130,Melbi2_572993,Rhier1_600383,HypCI4A1_321098,HypCO2751_17521,HypEC381_395331,Melbi2_696696,Phisc1_706092,Cadsp1_350273,Glalo1_5527,Clagr3_4470,Aspzo1_105929,Aspzo1_26514,Aurpuvarpul1_291524,Aurpuvarsub1_102588,Aurpuvarmel1_13202,Aurpuvarnam1_86426,Parbr1_3036,Bder_01754T0,Hisca1_5375,Talma12_829,Pyrco1_5592,Morco1_524827,Morimp1_550840,Aspgl1_119608,Eurhe1_414300,Penox1_2509,Pendi1_5483,Pench1_76464,PenchWisc11_140476,Aspwe1_26209,Aspcl1_3624,Aspfu1_7733,AspfuA11631_106205,Aspnov1_404651,Neofi1_9562,Aspnid1_10092,Aspve1_133004,Aspor1_2736,Aspoch1_461782,Aspac1_28147,Aspca3_404216,Aspca3_45444,Asptu1_49841,Aspfo1_63878,Aspka11_21515,Aspbr1_57866,AspniDSM1_158324,Aspni7_1186485,AspniNRRL31_8553),(Amapo0_3172.1,Amabr0_14327.1,Amabr0_7709.1,Amamu0_12797.1,Amamu0_12806.1,Amamu0_7561.1,Pucgr1_23743,Rhosp1_3647,Schco3_2489244,Armme11_2344,Exigl1_615646,Trave1_110824,Tilan2_249267,Punst1_124096,Helsul1_1629573,Neole1_1054711,Dacsp1_108877,Calco1_463966,Calvi1_126822,Sebve1_61343,Tulca1_220580,Sphst1_168580,Phaca1_256164,Phchr2_3001131,Fomme1_145373,Dicsq1_177432,Armme11_2624,Galma1_59305,Klula1_3451,Ascru1_74553,Calb_04312,Picst3_44393,Ppat1_XP_001755806.1,Pabi1_MA_362355g0010,Psme1_PSME_00030339-RA,Egui1_XP_010925634.1,Pdac1_XP_008789985.1,Cari1_XP_004514648.1,Cari1_XP_004514650.1,Natt1_XP_019249491.1,Nsyl1_XP_009798852.1,Ntab1_XP_016456661.1,Bnap1_XP_013736281.1,Brap1_XP_009135412.1,Garb1_XP_017617963.1,Ghir1_XP_016676131.1,Pila1_PILAlq_041310-RA,Pita1_PITA_000037874-RA,Atau1_XP_020151510.1,Sbic1_XP_002441054.1,Egui1_XP_010921004.1,Dcat1_XP_020685699.1,Pequ1_XP_020585972.1,Bvul2_XP_010693058.1,Inil1_XP_019151362.1,Cann1_XP_016547742.1,Cann1_XP_016564113.1,Natt1_XP_019230611.1,Ntom1_XP_009631854.1,Ntab1_XP_016471998.1,Ntom1_XP_009605678.1,Natt1_XP_019258060.1,Nsyl1_XP_009765120.1,Ntab1_XP_016475074.1,Jcur1_XP_012086876.1,Garb1_XP_017643464.1,Ghir1_XP_016681672.1,Mtru1_XP_003616684.1,Ccaj1_XP_020225890.1,Vang1_XP_017430394.1,Vrad1_XP_014504725.1,Jreg1_XP_018821797.1,Alyr1_XP_002873210.1,Atha1_NP_568155.1,Crub1_XP_006290079.1,Csat1_XP_010452449.1,Csat1_XP_010491079.1,Esal1_XP_006399009.1,Rsat1_XP_018476869.1,Rsat1_XP_018476870.1,Bole1_XP_013628953.1,Bnap1_XP_013724111.1,Brap1_XP_009130883.1));

**Supplementary file 4**

**Supplementary file 6**

**Supplementary file 7**

**
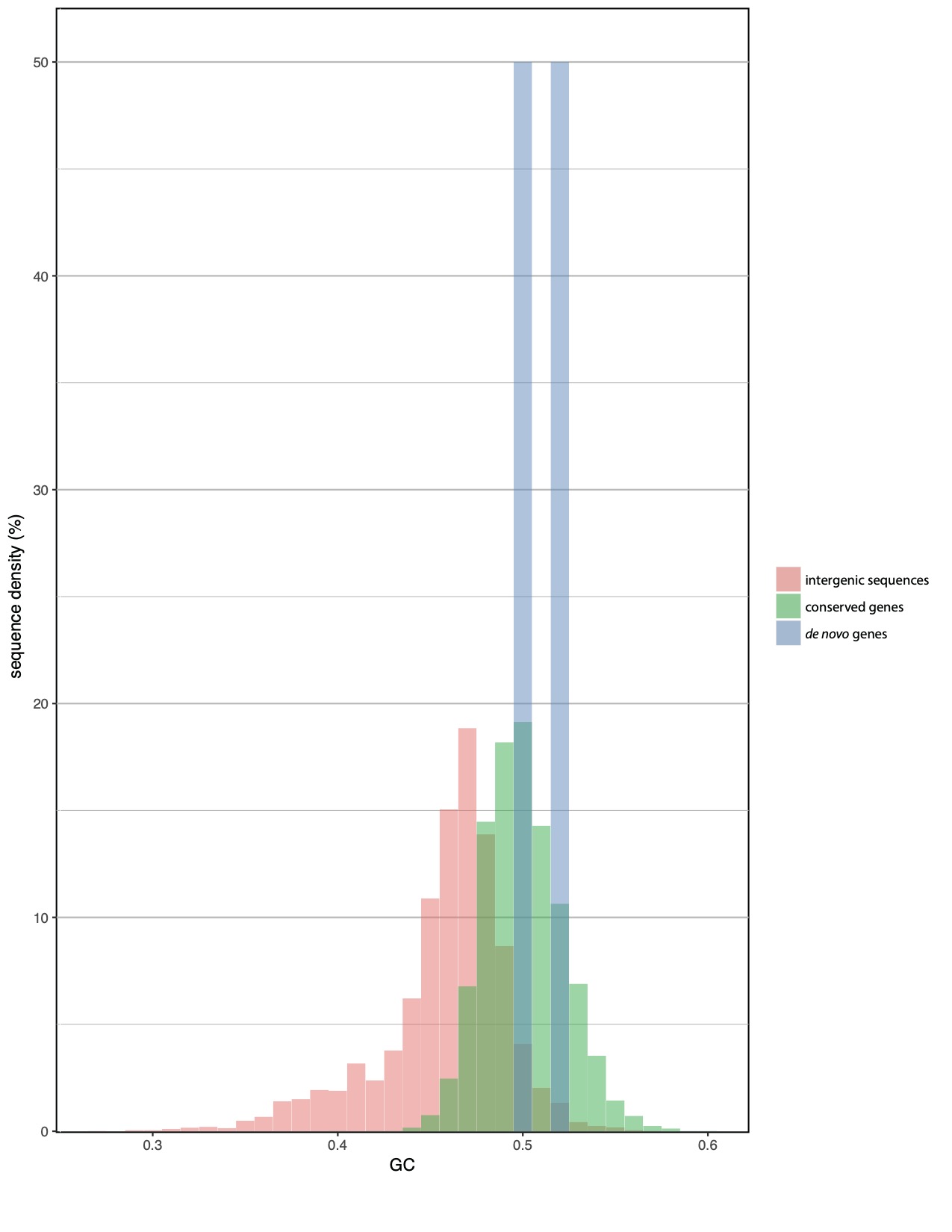
**

**Supplementary file 8**

**
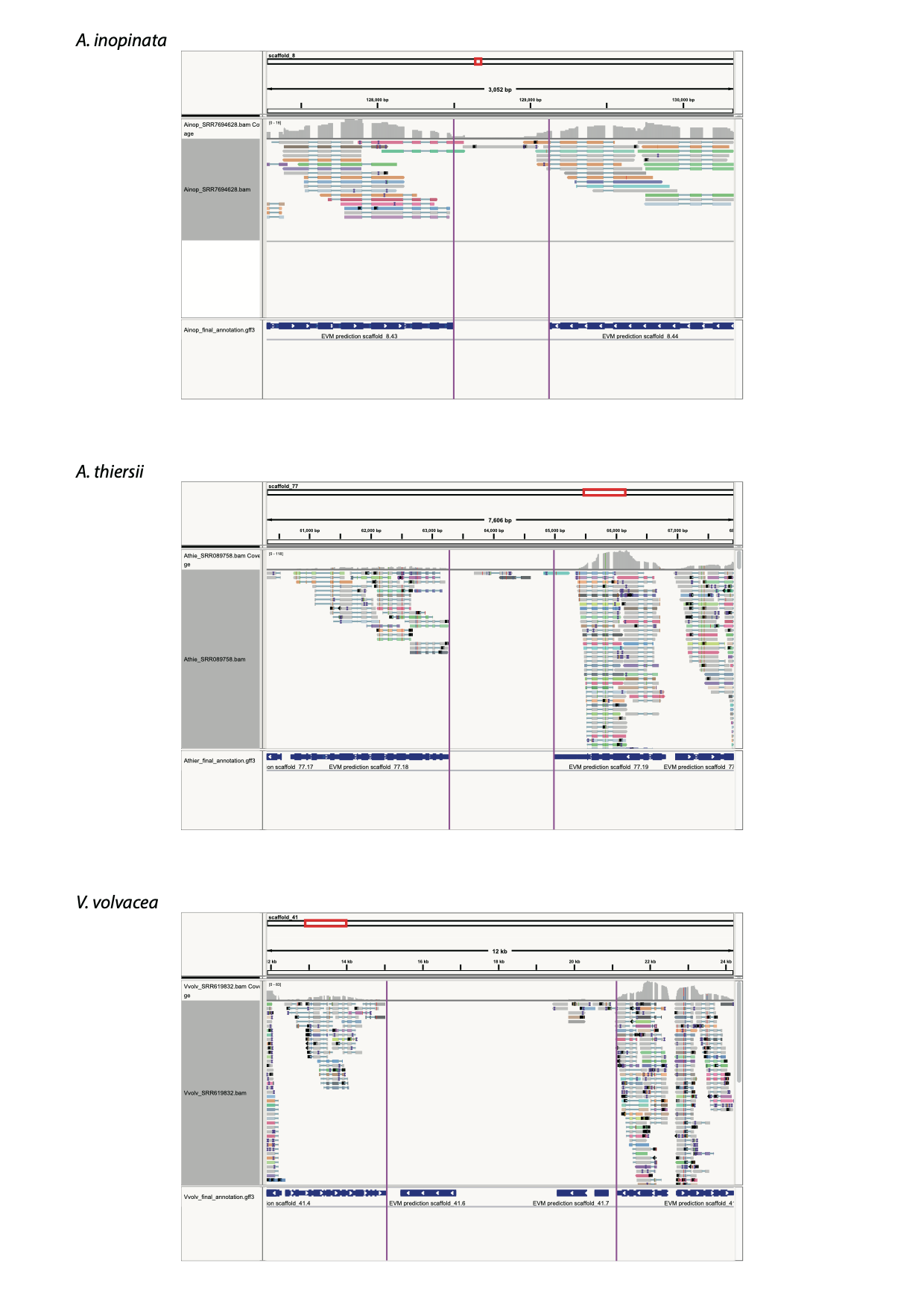
**
