# Supplementary material for: De Novo Gene Birth, Horizontal Gene Transfer, and Gene Duplication as Sources of New Gene Families Associated with the Origin of Symbiosis in Amanita
Source: Genome Biol Evol. 2020 Sep 14;12(11):2168–82. doi: 10.1093/gbe/evaa193 (PMC7674699; doi:10.1093/gbe/evaa193)
Supplement: evaa193_Supplementary_Data [file evaa193_supplementary_data.zip › SupplementaryLegends.docx]

Supplementary file legends

**Supplementary file 1**

Fungal genomes in the database used in this study

**Supplementary file 2**

Plant genomes in the database used in this study

**Supplementary file 3**

Constraint trees for AU test to reject vertical transmission.

**Supplementary file 4**

The number of total families or families unique to ECM *Amanita* while using different inflation number for FastOrtho.

**Supplementary file 5**

The inferred origin, dN/dS ratio and GO terms of the families unique to ECM *Amanita*. “Extended de novo”: families with no homolog found in our curated database but with homologs found in GenBank or UniProt database; “Extended HGT”: a putative HGT family not passing any AU tests; “Extended duplication”: putative duplicated families without bootstrap value ≥ 80 at the duplicating branches or having other species clustering with ECM *Amanita*; “Extended selective retention”: families with homologs but without orthologs in the three asymbiotic species. AMGO, ABGO and APGO are all the GO terms of all the genes of a specific family from *A. muscaria* var. *guessowii*, *A. brunnescens* and *A. polypyramis*. Shared GO is the GO terms shared by AMGO, ABGO and APGO.

**Supplementary file 6**

The ratio of the number of genes upregulated in axenic culture to the total number of genes for conserved (n=5264), unique (n=272) or orphan families (n=4989). Different fold cutoffs (2, 4 and 8) are used to define upregulation in subplots A, B and C. Fisher exact test P-value < 10^-4^ for all cutoffs. Letters mark significant (adjusted P-value < 0.05).

**Supplementary file 7**

Density frequency histogram of the GC content of intergenic sequences, conserved genes and *de novo* genes.

**Supplementary file 8**

Transcriptomic raw reads mapping to the non-coding region homologous to *de novo* family 3446 in the three asymbiotic species. Purple vertical lines specify the ends of the homologous region.

**Supplementary file 9**

P-values of AU tests for putative HGT gene families with different constraints (see Supplementary file 3). Low P-values for constraint trees reject the hypothesis of vertical transmission.
